# A Specific *CNOT1* Mutation Results in a Novel Syndrome of Pancreatic Agenesis and Holoprosencephaly through Impaired Pancreatic and Neurological Development

**DOI:** 10.1016/j.ajhg.2019.03.018

**Published:** 2019-04-18

**Authors:** Elisa De Franco, Rachel A. Watson, Wolfgang J. Weninger, Chi C. Wong, Sarah E. Flanagan, Richard Caswell, Angela Green, Catherine Tudor, Christopher J. Lelliott, Stefan H. Geyer, Barbara Maurer-Gesek, Lukas F. Reissig, Hana Lango Allen, Almuth Caliebe, Reiner Siebert, Paul Martin Holterhus, Asma Deeb, Fabrice Prin, Robert Hilbrands, Harry Heimberg, Sian Ellard, Andrew T. Hattersley, Inês Barroso

**Affiliations:** 1 Institute of Biomedical and Clinical Science, University of Exeter Medical School, EX2 5DW Exeter, UK; 2 Wellcome Sanger Institute, CB10 1SA Hinxton, UK; 3 Centre for Anatomy and Cell Biology & MIC, Medical University of Vienna, 1090 Vienna, Austria; 4 Institute of Human Genetics, Christian-Albrechts-University 24105 Kiel and University Hospital Schleswig-Holstein, 24105 Kiel, Germany; 5 Institute of Human Genetics, Ulm University & Ulm University Medical Center, 89081 Ulm, Germany; 6 Department of Pediatrics, Division of Pediatric Endocrinology and Diabetes, Christian-Albrechts-University 24105 Kiel and University Hospital Schleswig-Holstein, 24105 Kiel, Germany; 7 Paediatric Endocrinology Department, Mafraq Hospital, 2951 Abu Dhabi, United Arab Emirates; 8 The Francis Crick Institute, NW1 1ST London, UK; 9 Vrije Universiteit Brussel, 1090 Brussels, Belgium; 10 Universitair Ziekenhuis Brussel, 1090 Brussels, Belgium

**Keywords:** neonatal, diabetes, pancreas, agenesis, genetics, mutation, development, neurological

## Abstract

We report a recurrent *CNOT1 de novo* missense mutation, GenBank: NM_016284.4; c.1603C>T (p.Arg535Cys), resulting in a syndrome of pancreatic agenesis and abnormal forebrain development in three individuals and a similar phenotype in mice. CNOT1 is a transcriptional repressor that has been suggested as being critical for maintaining embryonic stem cells in a pluripotent state. These findings suggest that CNOT1 plays a critical role in pancreatic and neurological development and describe a novel genetic syndrome of pancreatic agenesis and holoprosencephaly.

## Main Text

Discovering genes with mutations causal of pancreatic agenesis is crucial to identifying factors needed for pancreatic development. To date, pathogenic variants in six genes (*PTF1A* [MIM: 615935], *PDX1* [MIM: 260370], *GATA6* [MIM: 600001], *GATA4* [MIM: 600576], *HNF1B* [MIM: 137920], and *RFX6* [MIM: 615710]) have been reported to severely affect pancreatic development and result in pancreatic agenesis.^1^ Gene discovery in pancreatic agenesis has shown both similarities and marked differences between pancreatic development in human and mouse. In both species, complete loss of function of *PTF1A*, *PDX1*, or *RFX6* results in pancreatic agenesis. In contrast, while haploinsufficiency of *GATA6* is a common cause of pancreatic agenesis in humans,^2^ in mice *Gata6* knockout does not result in abnormal pancreatic development.^3^ Knowledge of human pancreatic development is essential to guide progress of beta-cell replacement therapy for people with type 1 diabetes.

We investigated an international cohort of 107 individuals diagnosed with pancreatic agenesis—defined by requiring both endocrine (insulin) and exocrine (pancreatic enzymes) replacement therapy within the first 6 months of life—and identified a mutation in a known gene in 98 of them (Table S1). To identify *de novo* mutations in the remaining nine subjects, exome sequencing was performed for the probands and both their unaffected parents when available (n = 7) (Supplemental Subjects and Methods).

**Figure 1:**
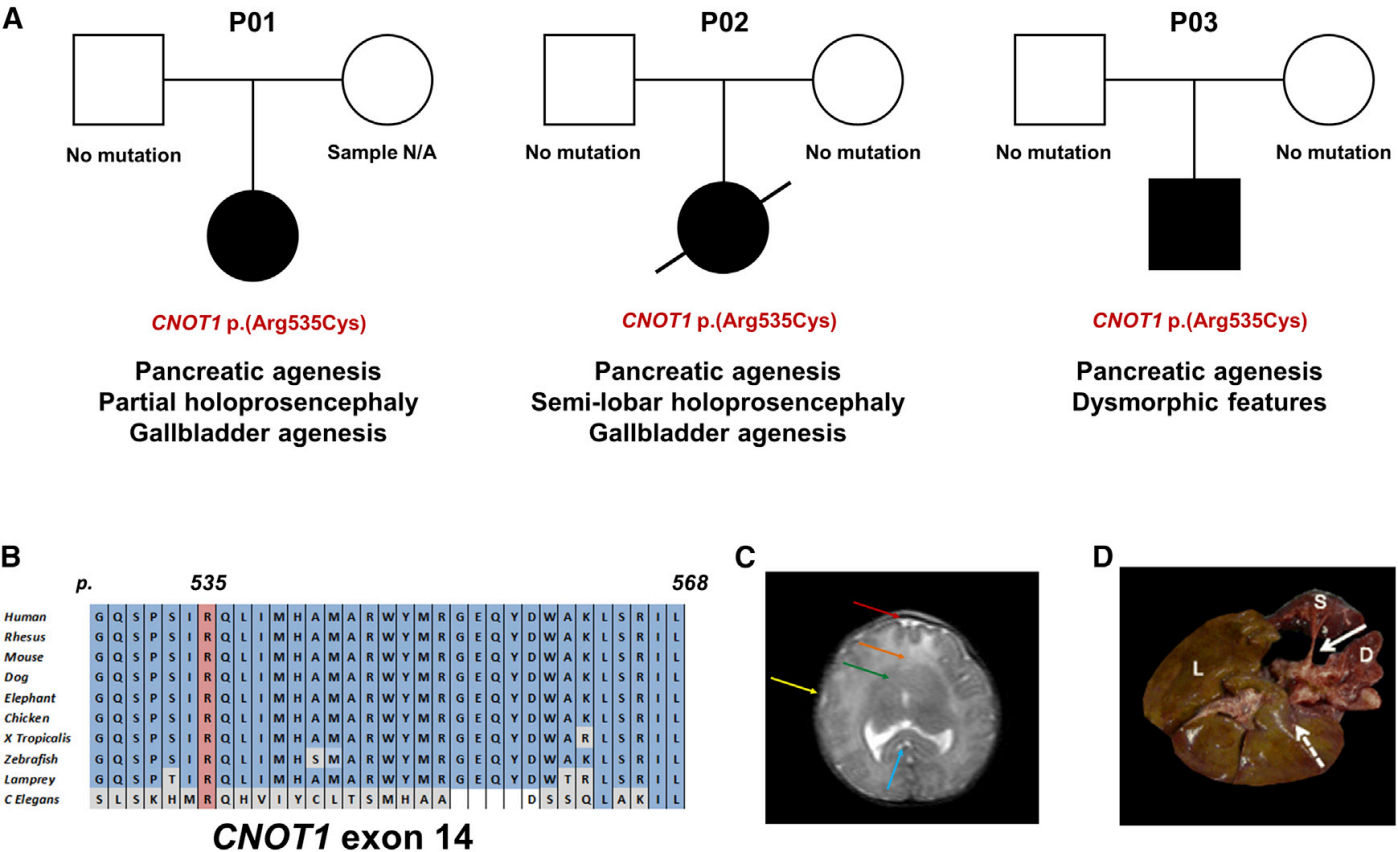
Genetic and Clinical Findings in Individuals with Pancreatic Agenesis (A) Partial pedigrees and clinical features of the three individuals with the heterozygous *CNOT1* p.Arg535Cys mutation. (B) Conservation of CNOT1 residues 529 to 568 across 10 representative species. Residue p.Arg535 is highlighted in red. Residues identical to the human CNOT1 protein are highlighted in blue, differences are highlighted in gray. (C) Coronal brain MRI of P02 showing absence of the anterior interhemispheric fissure (red arrow), fusion of the frontal lobes (orange arrow), absence of frontal horns (green arrow), absence of the sylvian fissures (yellow arrow). Splenium of the corpus callosum is visible (blue arrow). (D) Post mortem image of P02’s liver (L), spleen (S), and duodenum (D). White arrow shows the orthotopic location of the pancreas, which is absent. Dashed arrow indicated site in which the absent gallbladder would be expected to be.

We identified a heterozygous missense mutation in *CNOT1* (MIM: 604917; GenBank: NM_016284.4; c.1603C>T [p.Arg535Cys]) in three individuals with pancreatic agenesis. The variant had arisen *de novo* in two of them and was not present in the DNA sample from the 3^rd^ individual’s father (maternal sample was not available for testing) (Figure 1A, Tables S2 and S3). We confirmed these results by Sanger sequencing (Supplemental Subjects and Methods, Figure S1). The p.Arg535Cys variant is absent from dbSNP138, DECIPHER, and GnomAD and affects a residue which is highly conserved across species (up to *C. elegans*) (Figure 1B). All three *in silico* prediction tools used (AlignGVGD, PolyPhen2, and SIFT accessed through AlamutVisual) predicted the variant to have a deleterious effect on protein function (Supplemental Subjects and Methods).

The three individuals who were heterozygous for the *CNOT1* p.Arg535Cys variant had strikingly similar clinical features (see Supplemental Note). In addition to pancreatic agenesis, all three had definite (n = 2) or possible holoprosencephaly (Figure 1A, Table S4), a disorder in which the prosencephalon (forebrain of the embryo) fails to develop into two hemispheres. P01 and P02 (who was previously reported by Hilbrands et al.^4^) both had partial/semi-lobar holoprosencephaly, while P03 has dysmorphic features which could be consistent with holoprosencephaly (prominent central incisors and occiput, highly arched palate, and low-set ears) but brain MRI was declined by his parents and the diagnosis could not therefore be confirmed. All three individuals had very low birth weight (Z-score < −2), likely due to insulin deficiency in the last trimester of pregnancy, when insulin is the main fetal growth factor. Consistent with insulin deficiency *in utero*, the three case subjects all developed diabetes very early (2/3 diagnosed at 1 day and 1 at 13 weeks). P01 and P02 also had gallbladder agenesis, a clinical feature frequently associated with pancreatic agenesis.

The DDD study^5^ has identified *de novo CNOT1* variants in three individuals with developmental delay (two missense—p.Leu2323Phe and p.Arg623Trp—and a nonsense—p.Gln33^∗^—variant) but none of them had holoprosencephaly or diabetes. Since our three case subjects were all heterozygous for the same novel missense *CNOT1* variant and none of the DDD participants with heterozygous *de novo CNOT1* variants had pancreatic or neurological structural malformations, we hypothesized that a mutation-specific mechanism rather than loss of function was responsible for the phenotype seen in our case subjects. We therefore generated a mouse line harboring the *Cnot1*^p.(Arg535Cys)^ mutation using CRISPR (Supplemental Subjects and Methods).

**Figure 2:**
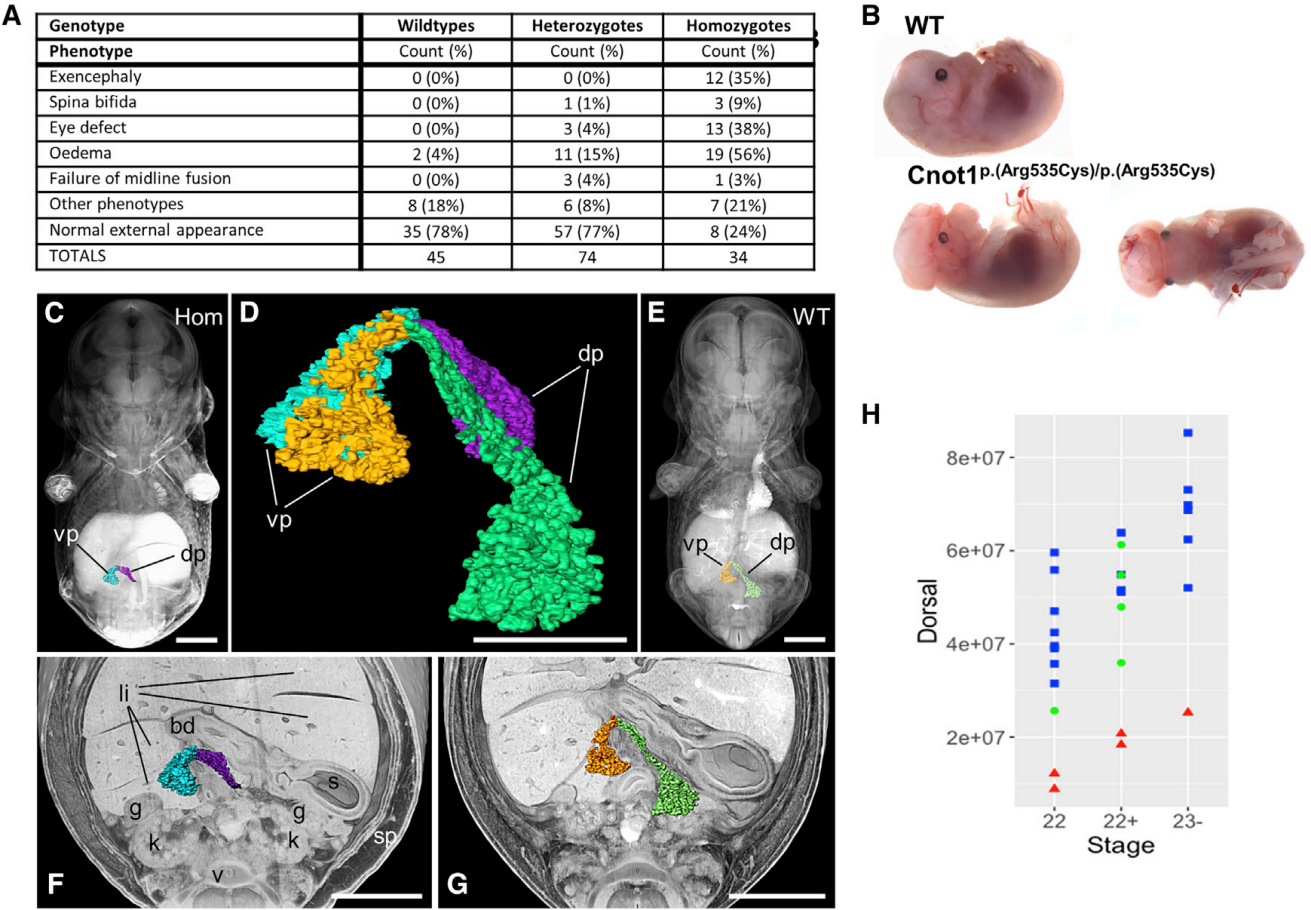
Neurological and Pancreatic Abnormalities in Mouse Embryos Homozygous for the *Cnot1* p.Arg535Cys Mutation (A) Table listing the gross external phenotypes observed in E14.5 embryos. Numbers do not add to total as many embryos displayed multiple phenotypes. Significance by Fisher’s exact test, assuming an additive model. Exencephaly, p = 3.2 × 10^−9^; spina bifida, p = 0.027; eye defect, p = 5.5 × 10^−8^; edema, p = 2.6 × 10^−7^; midline defect, ns. (B) Images showing representative E14.5 embryos: top shows wild-type embryo, bottom shows embryo homozygous for the *CNOT1* p.Arg535Cys mutation with exencephaly and coloboma. (C and E) Coronally sectioned, semi-transparent 3D volume models of stage-matched E14.5 embryos with superimposed models of the pancreas of homozygous (C) and wild-type (E) embryos. (D) Overlay of extracted surface models of the pancreas of homozygous (blue, magenta) and wild-type embryos (orange, green). (F and G) Coronally sectioned solid 3D volume rendered model of the abdomen of the embryos shown in (C) and (E) with superimposed pancreas. dp, dorsal pancreas; vp, ventral pancreas; li, liver lobes; s, stomach; sp, spleen; k, kidney; g, gonad; bd, bile duct. Scale bars: 1,000 μm in (C), (E)–(G); 500 μm in (D). (H) Graph showing the volume of the dorsal pancreas of E14.5 embryos in μm^3^. Blue squares show wild types, green circles are heterozygotes, and red triangles are homozygotes. Data analyzed using ANOVA with TukeyHSD posthoc test, effect of genotype p = 8.85 × 10^−8^; post hoc WT-Hom, p < 10^−10^; Het-Hom, p = 1.36 × 10^−4^, WT-Het, ns.

Heterozygous mice were born at a lower than expected frequency (Table S5), but without an obvious phenotype, while homozygosity for the mutation was embryonically lethal. At E14.5, embryos were still alive and present at expected Mendelian ratios and were therefore collected to assess their phenotype (Supplemental Subjects and Methods). Upon dissection, several gross morphological abnormalities were apparent in homozygotes, notably exencephaly, eye defects (mostly coloboma), and edema (Figures 2A, 2B, and S2; Table S6).

High-resolution episcopic microscopy (HREM) highlighted a significant reduction in the size of the pancreas in homozygous embryos in addition to several other abnormalities (Figures 2C–2G, Supplemental Subjects and Methods, DMDD website). The reduction in pancreatic size was found to be predominantly due to a smaller dorsal pancreas (Figures 2H and S3). These results provide compelling evidence for the role of *Cnot1*^p.(Arg535Cys)^ in pancreatic development.

**Figure 3:**
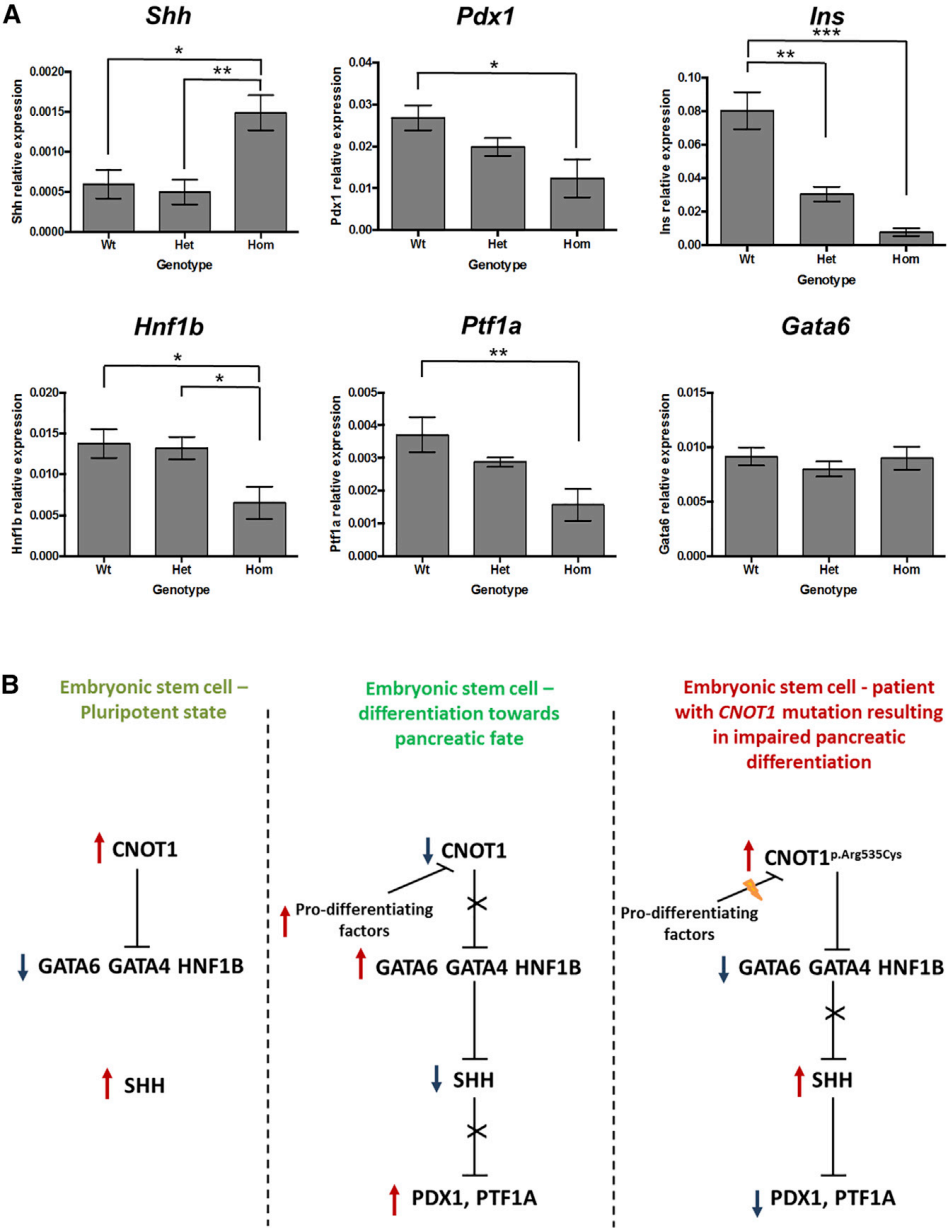
Expression Data and Possible Mechanism Involving CNOT1 in Pancreatic Development (A) Graphs showing relative expression of genes in the pancreas of E14.5 embryos. Bars show mean ± SE. Data analyzed using ANOVA with TukeyHSD posthoc test. Results of posthoc tests shown on graphs, ^∗^p < 0.05, ^∗∗^p < 0.01, ^∗∗∗^p < 0.001. *Shh*, effect of genotype p = 0.0107; *Pdx1*, effect of genotype p = 0.0189; *Ins*, effect of genotype p = 7.03 × 10^−6^; *Hnf1b*, effect of genotype p = 0.0294; *Ptf1a*, effect of genotype p = 0.00781; *Gata6*, effect of genotype p = ns. n = 4–12 animals per genotype. (B) Schematic representation of the proposed role for CNOT1 in pancreatic development.

Expression analysis of pancreatic developmental factors on RNA extracted from pancreatic tissue in E14.5 wild-type, *Cnot1*^p.(Arg535Cys)/p.(Arg535Cys)^, and *Cnot1*^p.(Arg535Cys)^ embryos showed a significant increase of *Shh* expression in homozygous embryos, with decreased expression in *Pdx1*, *Ins*, *Hnf1b*, and *Ptf1a* (Figure 3A). No difference in expression was detected for *Gata6* (Figure 3A) and *Rxra* (Figure S4).

The pancreatic and neurological phenotypes observed in *Cnot1*^p.(Arg535Cys)/p.(Arg535Cys)^ E14.5 mouse embryos are consistent with the pancreatic agenesis and holoprosencephaly observed in the three case subjects, confirming that the *de novo CNOT1* mutation is indeed the cause of their disease. Mice required a homozygous mutation in *Cnot1* to display a pancreatic and brain phenotype while a heterozygous *CNOT1* mutation resulted in the phenotype in three individuals in our cohort. This has been described with other pancreatic developmental genes (e.g., *HNF1B*) and supports the hypothesis that the early stages of pancreatic development are not identical in mice and humans.^6^

The CNOT1 protein has not previously been suggested to have a role in pancreatic development; it is known to act both as scaffold of the CCR4-NOT complex and as an independent factor. As such, it mediates transcriptional repression^7^ and is expressed extremely early during embryonic development (E3.5 in the inner cell mass in mice^8^). *In vitro* studies have proposed that CNOT1 plays a critical role in maintaining human and mice embryonic stem cells in a pluripotent state by inhibiting primitive endoderm factors.^8^ *CNOT1* expression peaks in undifferentiated human induced pluripotent stem (iPS) cells compared to subsequent stages of *in vitro* differentiation toward pancreatic endocrine cells,^9^ supporting its fundamental role in stem cells.

The increased expression of Shh in pancreatic tissue extracted from Cnot1^p.(Arg535Cys)/p.(Arg535Cys)^ embryos would be consistent with a model in which the *CNOT1* p.Arg535Cys mutation results in embryonic stem cells being maintained in an undifferentiated state through SHH-mediated inhibition of differentiation. SHH is a key developmental factor that is known to be crucial for pancreatic and brain development. Heterozygous loss-of-function mutations in *SHH* cause holoprosencephaly (MIM: 142945) and studies in both mouse and human embryos have shown that *SHH* expression needs to be repressed in the dorsal foregut endoderm for successful differentiation toward dorsal pancreas.^6^, ^10^ A recent study has suggested that the transcription factors Gata4 and Gata6 (mutations in which are a cause of pancreatic agenesis) regulate pancreatic endoderm identity by directly inhibiting Shh in mice.^11^ It is therefore possible that the p.Arg535Cys variant results in CNOT1 maintaining its inhibition activity on the GATA and other early differentiation factors and, as a consequence, SHH expression is not repressed (Figure 3B). Increased expression of *Shh* and decreased expression of *Pdx1*, *Ins*, *Hnf1b*, and *Ptf1a* detected in RNA extracted from pancreatic tissue in the E14.5 *Cnot1*^p.(Arg535Cys)/p.(Arg535Cys)^ embryos would support this hypothesis. However, Gata4 expression could not be assessed as the assay specificity was too low and Gata6 expression was not found to be reduced. It is possible that Gata6 activity is actually inhibited earlier during development and then re-activated by a different pathway (Gata6 is needed for development of most endodermal-derived organs and heart) or could be inhibited by a different mechanism that does not result in reduced expression. The *CNOT1* p.Arg535Cys mutation also affects neurological development in both our case subjects and mouse embryos. It is possible that this mutation results in ectopic SHH expression during brain development. This would be consistent with previous reports of Shh ectopic expression impairing midline development.^12^ Another possibility is that the effect of the *CNOT1* mutation on SHH signaling differs between the brain and the pancreas, resulting in a reduced expression in the developing brain and increased expression during pancreatic development. Further experiments, ideally on younger embryos and human iPS cells, are needed in order to elucidate the mechanism by which the *CNOT1* p.Arg535Cys mutation results in impaired pancreatic and neurological development.

Our study identifies a spontaneous *CNOT1* p.Arg535Cys mutation as the genetic cause of a rare syndrome of pancreatic agenesis and holoprosencephaly, highlighting a previously unsuspected role of CNOT1 as a key factor in both pancreatic and neurological development. This is the 7^th^ gene causative of pancreatic agenesis described so far and the first pancreatic agenesis gene that is thought to be important for maintaining stem cells’ pluripotency. These findings suggest a new mechanism by which impairment of the very early stages of development result in pancreatic agenesis and abnormal brain development.

## Declaration of Interests

The authors declare no competing interests.

## Web Resources

DECIPHER, https://decipher.sanger.ac.uk/
DMDD, https://dmdd.org.uk
GenBank, https://www.ncbi.nlm.nih.gov/genbank/
gnomAD Browser, https://gnomad.broadinstitute.org/
OMIM, http://www.omim.org/

## Supplemental Data

Document S1. Supplemental Note, Figures S1–S4, Tables S1–S6, and Supplemental Subjects and Methods

Document S2. Article plus Supplemental Data
