# Supplementary material for: A Specific CNOT1 Mutation Results in a Novel Syndrome of Pancreatic Agenesis and Holoprosencephaly through Impaired Pancreatic and Neurological Development
Source: Am J Hum Genet. 2019 Apr 18;104(5):985–9. doi: 10.1016/j.ajhg.2019.03.018 (PMC6506862; doi:10.1016/j.ajhg.2019.03.018)
Supplement: Document S1. Supplemental Note, Figures S1–S4, Tables S1–S6, and Supplemental Subjects and Methods [file mmc1.pdf]

The American Journal of Human Genetics, Volume *104*

Supplemental Data

A Specific *CNOT1* Mutation Results in a Novel Syndrome of Pancreatic Agenesis and Holoprosencephaly through Impaired Pancreatic and Neurological Development

Elisa De Franco, Rachel A. Watson, Wolfgang J. Weninger, Chi C. Wong, Sarah E. Flanagan, Richard Caswell, Angela Green, Catherine Tudor, Christopher J. Lelliott, Stefan H. Geyer, Barbara Maurer-Gesek, Lukas F. Reissig, Hana Lango Allen, Almuth Caliebe, Reiner Siebert, Paul Martin Holterhus, Asma Deeb, Fabrice Prin, Robert Hilbrands, Harry Heimberg, Sian Ellard, Andrew T. Hattersley, and Inês Barroso

Supplemental Data

Supplemental Note: Case reports

Case P01

P01 is a 13 years and 9 months-old girl born to non-consanguineous parents. She was born at 38+4 weeks gestation after a pregnancy complicated by growth retardation and corpus callosum agenesis detected sonographically. She was born small for gestational age (birth-weight 1340g, length 41 cm and OFC 30 cm). She developed insulin-dependent diabetes on day 1 of life. Abdominal ultrasound and MRI failed to detect pancreas and gallbladder, but the ductus choledochus was present and the intrahepatic gall ducts were normal. Gamma-glutamyl-transpeptidase was transiently elevated in the neonatal period (800 IU). Therapy with oral pancreatic enzymes for pancreatic exocrine insufficiency was commenced before discharge. Brain MRI confirmed a diagnosis of lobular holoprosencephaly with dysplastic frontal horns of the lateral ventricles, missing septum pellucidum, broadly joined cella media of the lateral ventricles, and hypoplasia of the corpus callosum (only present in the rostral portion of the corpus and the splenium, frontal parts not present). Muscle weakness, low-set ears and cardiac extrasystoly were also identified in the neonatal period. At the age of 3 years she developed complex focal seizures and she has been on sultiame therapy since. At the age of 3 years and one month, her height was 87cm (<3rd centile) and her weight was 11.6kg (between 3rd and 10th). At the age of 9 years she was reported to have mild learning difficulties. She is on growth hormone therapy (SGA-indication, pituitary growth hormone deficiency was excluded). Her current height is 152.2 cm (10th centile), weight 55kg (between 50th and 75th).

Case P02

P02 has been previously reported by Hilbrands *et al*[24]. Briefly, she was the second child of non-consanguineous parents. She was born at 38 weeks gestation, with a birth weight of 1100g, after an uneventful pregnancy complicated by intrauterine growth retardation. There was not family history of diabetes or brain anomalies. Dysmorphic features such as receding forehead, cylindrical nose, mild hypotelorism, dysplastic left ear, and hypoplastic zygomatic bone; and abducted thumbs were noted at birth. Diabetes was diagnosed in the first day of life and insulin treatment commenced. Exocrine insufficiency was confirmed during the second week of life and treatment with oral pancreatic enzymes was started. Complete absence of the pancreas and gallbladder were initially detected by abdomen MRI. Brain MRI confirmed semilobar holoprosencephaly with absent corpus callosum. She died at 12 weeks and 3 days.

Case P03

P03 was born after 39 weeks gestation and his birth weight was 1900g. His parents are not related and no family history of diabetes was reported. He was diagnosed with diabetes at the age of 3 weeks and treated with insulin. An abdominal ultrasound failed to detect a pancreas and a negative immune-reactive trypsin test confirmed exocrine pancreatic insufficiency, which was treated with oral pancreatic enzymes. Mild dysmorphic features (prominent occiput, low-set ears, high arched palate, prominent central incisors) and transient elevated liver enzymes (Alt 80 (NR 5-45), AST 66 (5-35), GGT 59 (3-22)) were reported. At last assessment (aged 16 years old) was still treated with oral pancreatic enzymes and insulin (dose 0.8U/kg/day on insulin pump). He was attending a normal school and there were no developmental concerns. A brain MRI scan was declined by his parents.

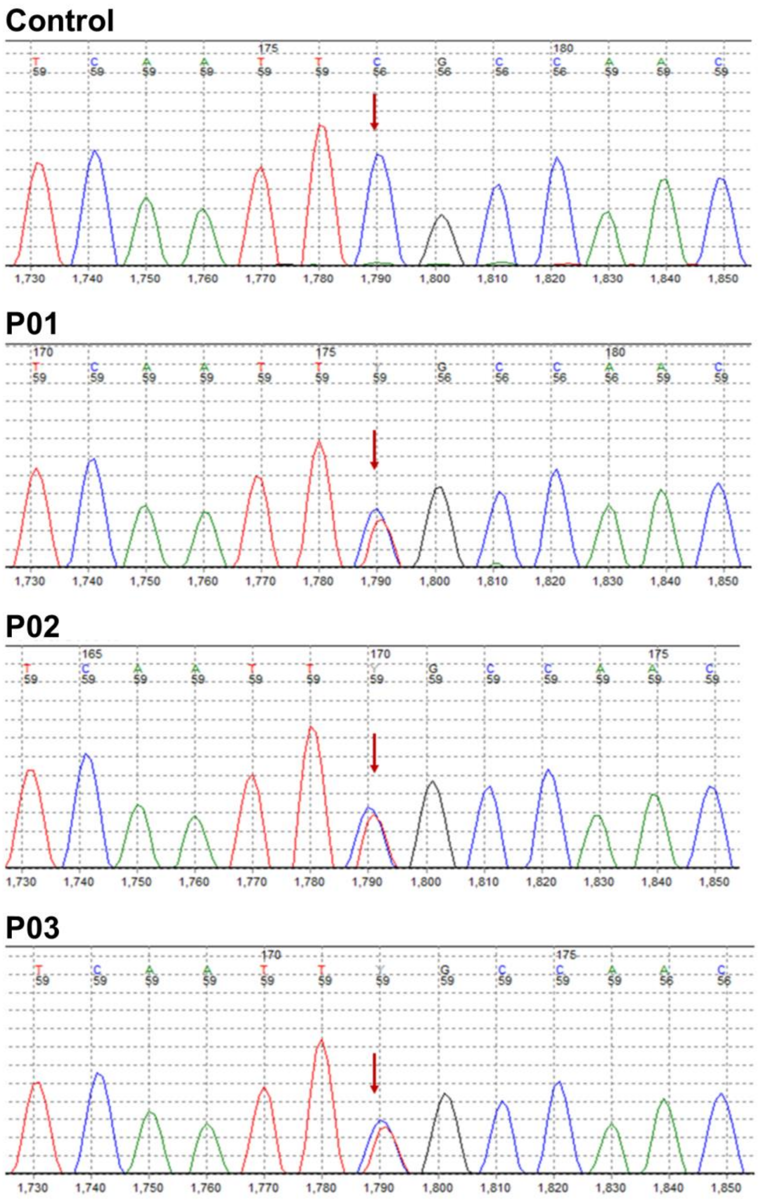

Figure S1. Sanger sequencing traces for the NM_016284.4(*CNOT1*):c.1603C>T, p.(Arg535Cys) variant (indicated by the red arrow).

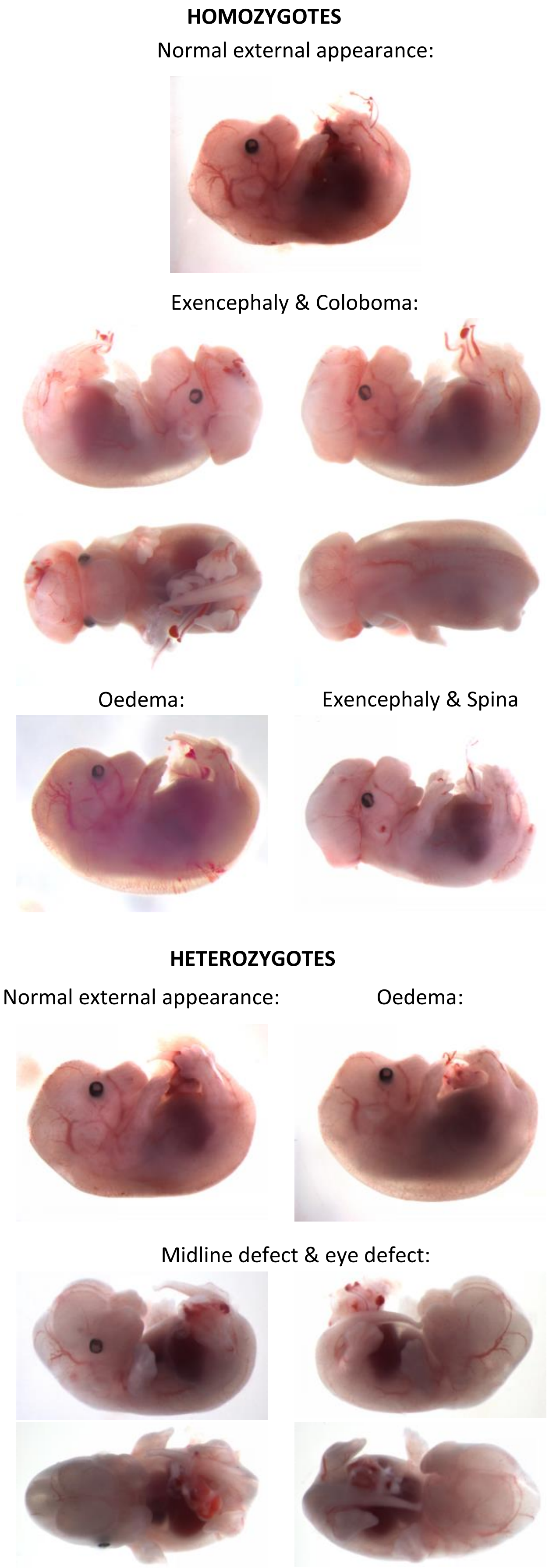

Figure S2. External morphology of E14.5 embryos.

A

Stage	Genotype	n	Dorsal pancreas volume (µm³)	Ventral pancreas volume (µm³)	Total pancreas volume (µm³)	Ratio Ventral:Dorsal
22	WT	6	39,205,654	16,695,357	55,901,011	0.42
	Het	1	25,633,746	12,693,213	38,326,959	0.50
	Hom	2	10,499,964	17,188,757	27,688,721	1.68
22+	WT	6	56,145,465	24,802,275	80,947,741	0.44
	Het	4	49,970,453	24,986,921	74,957,374	0.50
	Hom	2	19,544,099	20,346,290	39,890,389	1.05
23-	WT	6	68,546,795	32,482,914	101,029,709	0.48
	Het	0				
	Hom	1	25,231,986	30,382,804	55,614,790	1.20

B **C** **D**

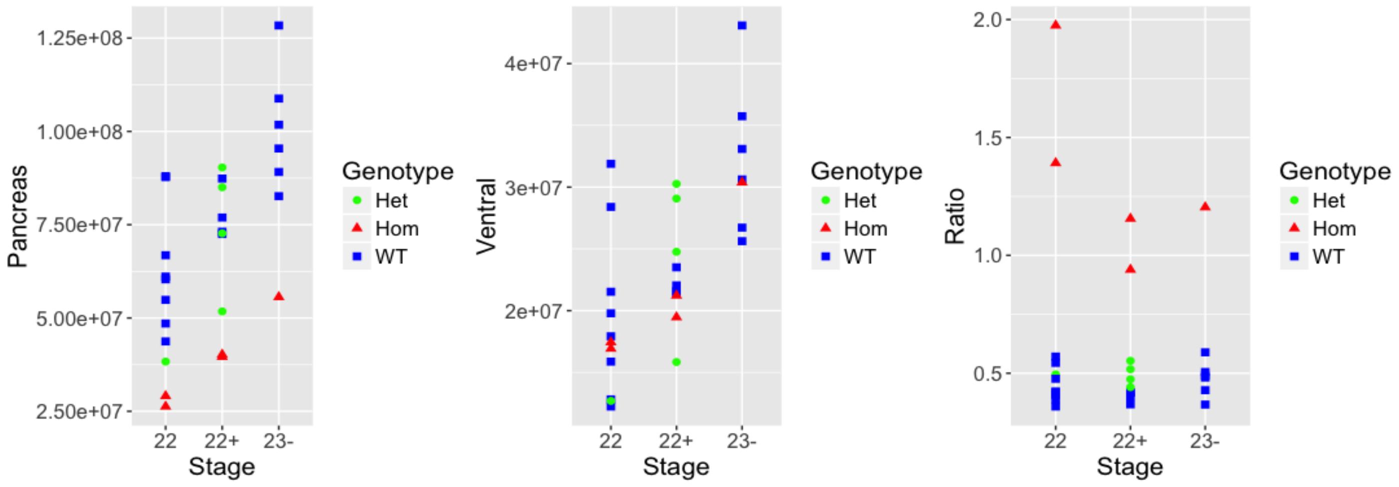

Figure S3. Pancreas size is reduced in E14.5 mutant embryos

A: Table showing the mean data for the pancreas volume determination.

B & C. Graphs showing the volume of the total (A) and ventral (B)pancreas in µm^3. Data analysed using ANOVA with TukeyHSD posthoc test. B: effect of genotype p=2.38x10^{-5}; post-hoc WT-Hom, p=1.46x10^{-5}; Het-Hom, p=0.007, WT-Het, ns. C: effect of genotype: ns.

D. Ratio of ventral:dorsal pancreas volume. Data analysed using ANOVA with TukeyHSD posthoc test; effect of genotype p=5.6x10^{-10}; post-hoc WT-Hom, p<10^{-10}; Het-Hom, p=1x10^{-7}, WT-Het, ns

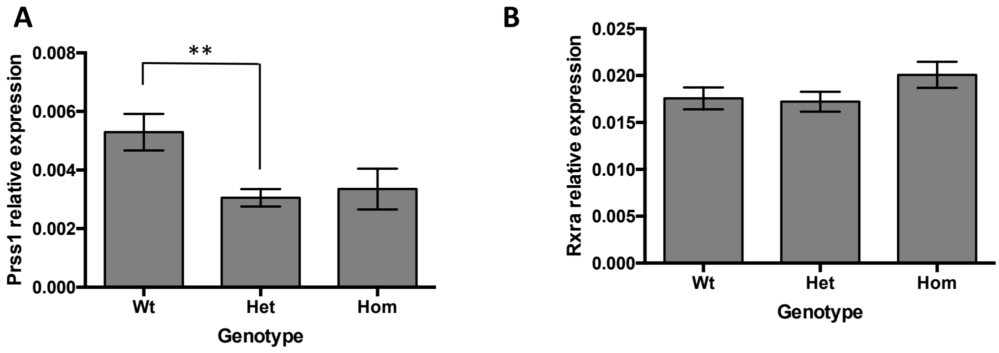

Figure S4. Relative expression of genes in the pancreas of E14.5 embryos. Bars show mean +/- SE. Data analysed using ANOVA with TukeyHSD posthoc test. Results of posthoc tests shown on graphs, * p<0.05, ** p<0.01, *** p<0.001. A: Prss1; effect of genotype p=0.00612. B. Rxra; effect of genotype ns.

ID	gene	Transcript	Nucleotide Change	Mutation Name	Exon Intron	Reference if previously reported
P01	*CNOT1*	NM_016284.4	c.1603C>T	p.(Arg535Cys)	Exon 14	
P02	*CNOT1*	NM_016284.4	c.1603C>T	p.(Arg535Cys)	Exon 14	
P03	*CNOT1*	NM_016284.4	c.1603C>T	p.(Arg535Cys)	Exon 14	
P04	*GATA4*	NM_002052.3	c.819C>A	p.(Asn273Lys)	Exon 4	[10]
P05	*GATA4*	NM_002052.3	c.(?_-554)_(1329+2_?)del	p.?	Exon 1-7	[10]
P06	*GATA4*	NM_002052.3	c.(?_-554)_(1329+2_?)del	p.?	Exon 1-7	
P07	*GATA6*	NM_005257.5	c.(?_-265)_(1135+2_?)del	p.?	Exon 1-2	
P08	*GATA6*	NM_005257.5	c.(?_-37)_(1788+2_?)del	p.?	Exon 1-7	
P09	*GATA6*	NM_005257.5	c.(?_-1)_(1788+2_?)del	p.?	Exon 1-7	
P10	*GATA6*	NM_005257.5	c.635_660del	p.(Pro212fs)	Exon 2	
P11	*GATA6*	NM_005257.5	c.701delC	p.(Pro234fs)	Exon 2	[11]
P12	*GATA6*	NM_005257.5	c.744delG	p.(Pro249fs)	Exon 2	[12]
P13	*GATA6*	NM_005257.5	c.877_880delinsTAC	p.(Val293fs)	Exon 2	
P14	*GATA6*	NM_005257.5	c.969C>A	p.(Tyr323*)	Exon 2	[13]
P15	*GATA6*	NM_005257.5	c.1013C>A	p.(Ser338*)	Exon 2	
P16	*GATA6*	NM_005257.5	c.1036_1042del	p.(Thr346fs)	Exon 2	[13]
P17	*GATA6*	NM_005257.5	c.1108_1121dup	p.(Glu375fs)	Exon 2	[11]
P18	*GATA6*	NM_005257.5	c.1136-2A>G	p.?	Intron 2	[13,14]
P19	*GATA6*	NM_005257.5	c.1242C>A	p.(Cys414*)	Exon 3	
P20	*GATA6*	NM_005257.5	c.1296del	p.(Lys432fs)	Exon 3	[15]
P21	*GATA6*	NM_005257.5	c.1303-10C>G	p.?	Intron 3	[11]
P22	*GATA6*	NM_005257.5	c.1303-2A>G	p.?	Intron 4	
P23	*GATA6*	NM_005257.5	c.1303-1G>T	p.?	Intron 4	[13]
P24	*GATA6*	NM_005257.5	c.1330T>C	p.(Cys444Arg)	Exon 4	
P25	*GATA6*	NM_005257.5	c.1354A>G	p.(Thr452Ala)	Exon 4	[11]
P26	*GATA6*	NM_005257.5	c.1366C>T	p.(Arg456Cys)	Exon 4	[11]
P27	*GATA6*	NM_005257.5	c.1366C>T	p.(Arg456Cys)	Exon 4	
P28	*GATA6*	NM_005257.5	c.1367G>A	p.(Arg456His)	Exon 4	[11]
P29	*GATA6*	NM_005257.5	c.1369A>G	p.(Arg457Gly)	Exon 4	
P30	*GATA6*	NM_005257.5	c.1396A>G	p.(Asn466Asp)	Exon 4	
P31	*GATA6*	NM_005257.5	c.1397A>G	p.(Asn466Ser)	Exon 4	[15,16]
P32	*GATA6*	NM_005257.5	c.1399G>A	p.(Ala467Thr)	Exon 4	[11]
P33	*GATA6*	NM_005257.5	c.1406G>A	p.(Gly469Glu)	Exon 4	[13]
P34	*GATA6*	NM_005257.5	c.1417A>C	p.(Lys473Gln)	Exon 4	[11]
P35	*GATA6*	NM_005257.5	c.1429-41_1441del	p.?	Intron 4	[13]
P36	*GATA6*	NM_005257.5	c.1429-8T>G	p.?	Intron 4	[13]
P37	*GATA6*	NM_005257.5	c.1435A>G	p.(Arg479Gly)	Exon 5	[13]
P38	*GATA6*	NM_005257.5	c.1448_1455del	p.(Met483fs)	Exon 5	[11,15]
P39	*GATA6*	NM_005257.5	c.1498_1501del	p.(Lys500fs)	Exon 5	[11]
P40	*GATA6*	NM_005257.5	c.1516+1G>C	p.?	Intron 5	[11]
P41	*GATA6*	NM_005257.5	c.1516+4A>G	p.?	Intron 5	[11]
P42	*PDX1*	NM_000209.3	c.455C>G/c.455C>G	p.(Ala152Gly)/p.(Ala152Gly)	Exon 2	[17,18]
P43	*PDX1*	NM_000209.3	c.478C>A/c.478C>A	p.(Glu160Lys)/p.(Glu160Lys)	Exon 2	
P44	*PDX1*	NM_000209.3	c.488A>G/c.488A>G	p.(Lys163Arg)/p.(Lys163Arg)	Exon 2	
P45	*PDX1*	NM_000209.3	c.518G>C/c.518G>C	p.(Arg173Pro)/p.(Arg173Pro)	Exon 2	
P46	*PDX1*	NM_000209.3	c.524G>T/c.524G>T	p.(Arg175Leu)/p.(Arg175Leu)	Exon 2	
P47	*PTF1A*	NM_178161.2	c.1A>G/c.1A>G	p.(Met1?)/p.(Met1?)	Exon 1	
P48	*PTF1A*	NM_178161.2	c.399dup/c.399dup	p.(Pro134fs)/p.(Pro134fs)	Exon 1	
P49	*PTF1A*	NM_178161.2	c.437_462del/g.23508442A>G	p.(Ala146fs)/p.?	Exon 1/Enhancer	[19]
P50	*PTF1A*	NM_178161.2	c.571C>A/c.571C>A	p.(Pro191Thr)p.(Pro191Thr)	Exon 1	[20]

P51	*PTF1A*	NM_178161.2	c.571C>A/c.571C>A	p.(Pro191Thr)p.(Pro191Thr)	Exon 1	[20]
P52	*PTF1A*	NM_178161.2	c.571C>A/c.571C>A	p.(Pro191Thr)p.(Pro191Thr)	Exon 1	
P53	*PTF1A*	NM_178161.2	c.571C>A/c.571C>A	p.(Pro191Thr)p.(Pro191Thr)	Exon 1	
P54	*PTF1A*	NM_178161.2	c.571C>A/c.571C>A	p.(Pro191Thr)p.(Pro191Thr)	Exon 1	
P55	*PTF1A*	NM_178161.2	c.784+4A>G/c.784+4A>G	p.?/p.?	Intron 1	[18]
P56	*PTF1A*	NM_178161.2	c.886C>T/c.886C>T	p.(Arg296*)/p.(Arg296*)	Exon 2	
P57	*PTF1A*		g.23508124-?_23508633+?del/g.23508124-?_23508633+?	p.?/p.?	Enchancer	[21]
P58	*PTF1A*		g.23508124-?_23508633+?del/g.23508124-?_23508633+?	p.?/p.?	Enchancer	
P59	*PTF1A*		g.23508124-?_23508633+?del/g.23508124-?_23508633+?	p.?/p.?	Enchancer	
P60	*PTF1A*		g.23508305A>G/g.23508305A>G	p.?/p.?	Enchancer	[21]
P61	*PTF1A*		g.23508336G>T/g.23508336G>T	p.?/p.?	Enchancer	
P62	*PTF1A*		g.23508363A>G/g.23508363A>G	p.?/p.?	Enchancer	[21]
P63	*PTF1A*		g.23508363A>G/g.23508363A>G	p.?/p.?	Enchancer	[22]
P64	*PTF1A*		g.23508363A>G/g.23508363A>G	p.?/p.?	Enchancer	
P65	*PTF1A*		g.23508363A>G/g.23508363A>G	p.?/p.?	Enchancer	
P66	*PTF1A*		g.23508363A>G/g.23508363A>G	p.?/p.?	Enchancer	
P67	*PTF1A*		g.23508363A>G/g.23508363A>G	p.?/p.?	Enchancer	
P68	*PTF1A*		g.23508363A>G/g.23508363A>G	p.?/p.?	Enchancer	
P69	*PTF1A*		g.23508363A>G/g.23508363A>G	p.?/p.?	Enchancer	
P70	*PTF1A*		g.23508363A>G/g.23508363A>G	p.?/p.?	Enchancer	
P71	*PTF1A*		g.23508365A>G/g.23508365A>G	p.?/p.?	Enchancer	
P72	*PTF1A*		g.23508365A>G/g.23508446A>C	p.?/p.?	Enchancer	[21]
P73	*PTF1A*		g.23508437A>G/g.23508437A>G	p.?/p.?	Enchancer	[21]
P74	*PTF1A*		g.23508437A>G/g.23508437A>G	p.?/p.?	Enchancer	[21]
P75	*PTF1A*		g.23508437A>G/g.23508437A>G	p.?/p.?	Enchancer	[21]
P76	*PTF1A*		g.23508437A>G/g.23508437A>G	p.?/p.?	Enchancer	[21]
P77	*PTF1A*		g.23508437A>G/g.23508437A>G	p.?/p.?	Enchancer	[21]
P78	*PTF1A*		g.23508437A>G/g.23508437A>G	p.?/p.?	Enchancer	[21]
P79	*PTF1A*		g.23508437A>G/g.23508437A>G	p.?/p.?	Enchancer	[22]
P80	*PTF1A*		g.23508437A>G/g.23508437A>G	p.?/p.?	Enchancer	
P81	*PTF1A*		g.23508437A>G/g.23508437A>G	p.?/p.?	Enchancer	
P82	*PTF1A*		g.23508437A>G/g.23508437A>G	p.?/p.?	Enchancer	
P83	*PTF1A*		g.23508437A>G/g.23508437A>G	p.?/p.?	Enchancer	
P84	*PTF1A*		g.23508437A>G/g.23508437A>G	p.?/p.?	Enchancer	
P85	*PTF1A*		g.23508437A>G/g.23508437A>G	p.?/p.?	Enchancer	
P86	*PTF1A*		g.23508437A>G/g.23508437A>G	p.?/p.?	Enchancer	
P87	*PTF1A*		g.23508437A>G/g.23508437A>G	p.?/p.?	Enchancer	
P88	*PTF1A*		g.23508437A>G/g.23508437A>G	p.?/p.?	Enchancer	
P89	*PTF1A*		g.23508437A>G/g.23508437A>G	p.?/p.?	Enchancer	
P90	*PTF1A*		g.23508437A>G/g.23508437A>G	p.?/p.?	Enchancer	
P91	*PTF1A*		g.23508437A>G/g.23508437A>G	p.?/p.?	Enchancer	
P92	*PTF1A*		g.23508437A>G/g.23508437A>G	p.?/p.?	Enchancer	
P93	*PTF1A*		g.23508437A>G/g.23508437A>G	p.?/p.?	Enchancer	
P94	*PTF1A*		g.23508437A>G/g.23508437A>G	p.?/p.?	Enchancer	
P95	*PTF1A*		g.23508437A>G/g.23508437A>G	p.?/p.?	Enchancer	
P96	*PTF1A*		g.23508437A>G/g.23508437A>G	p.?/p.?	Enchancer	
P97	*PTF1A*		g.23508437A>G/g.23508437A>G	p.?/p.?	Enchancer	
P98	*PTF1A*		g.23508437A>G/g.23508437A>G	p.?/p.?	Enchancer	
P99	*PTF1A*		g.23508437A>G/g.23508437A>G	p.?/p.?	Enchancer	
P100	*RFX6*	NM_173560.3	c.541C>T/c.541C>T	p.(Arg181Trp)/p.(Arg181Trp)	Exon 4	[23]
P101	*RFX6*	NM_173560.3	c.1573C>T/c.1573C>T	p.(Arg525*)/p.(Arg525*)	Exon 15	

Table S1. Pathogenic variants identified in the pancreatic agenesis cohort

Gene	gNomen	cNomen	pNomen	GnomAD Frequency	GnomAD pLI	GnomAD Z-sense (missense)
CNOT1	Chr16(GRCh37):g.58610468G>A	NM_016284.3:c.1603C>T	p.(Arg535Cys)	0%	1	7.62
SCAP	Chr3(GRCh37):g.47469072C>A	NM_012235.3:c.496G>T	p.(Glu166*)	0%	0	2.69
ZNF189	Chr9(GRCh37):g.104171066C>G	NM_003452.2:c.1016C>G	p.(Thr339Ser)	0%	0	0.51

Table S2. ***De novo*** **coding variants identified in P03**

ID	Genotype	Allele Depth	Read Depth
**P01**	0/1	28,38	63
**P02**	0/1	32,42	74
**P03**	0/1	30,15	45

Table S3. Exome sequencing allele and read depth of the NM_016284.4(*CNOT1*):c.1603C>T, p.(Arg535Cys) variant

ID	P01	P02[24]	P03
Birth weight (g)/ Gestation (weeks)	1340gr/39 wks (Z score=-3.5)	1100gr/38 wks (Z score=-3.8)	1900gr/39 wks (Z score=-2.4)
Gender	Female	Female	Male
CNOT1 Mutation	p.(Arg535Cys)	p.(Arg535Cys)	p.(Arg535Cys)
De novo	Maternal Sample N/A	Yes	Yes
Exome sequencing strategy	Singleton	Singleton	Trio
Age at last assessment (years)	11	Died at 12 weeks	18
Diabetes	Yes	Yes	Yes
Age at diabetes diagnosis	1 day	1 day	13 weeks
Insulin dose	On insulin pump (dose unknown)	N/A	0.9 u/kg/d
HbA1c	6.90%	N/A	8.30%
Pancreatic agenesis	Yes, (MRI)	Yes, (MRI and post mortem)	Abdominal CT
Exocrine pancreatic insufficiency	Yes, Creon treated	Yes, Creon treated	Yes, Creon treated
Gallbladder agenesis	Yes	Yes	
Neurological features	Lobular holoprosencephaly with dysplastic frontal horns of the lateral ventricles, missing septum pellucidum, broadly joined cella media of the lateral ventricles, malformation of the corpus callosum (only present in the rostral portion of the corpus and the splenium, the further frontal parts are not present). Complex focal seizures diagnosed aged 3 years.	Semi-lobar holoprosencephaly with polymicrogyria and fusion of the frontal lobes, absent corpus callosum	N/A
Developmental delay	Yes (mild), mainstream school at the age of 9 years with an accompanying person	N/A	No
Dysmorphic features	Low-set ears	Receding forehead, cylindrical nose, mild hypotelorism, dysplastic left ear, hypoplastic zygomatic bone	Craniofacial anomalies (prominent occiput, low-set ears, high arched palate, prominent central incisors)
Additional features	Transient elevation of LFTs, on growth hormone therapy	Thumbs in abduction at birth	Transient elevation of LFTs

Table S4. Clinical features of the patients with the *CNOT1* p.(Arg535Cys) mutation

Sex	Male		Female	
Genotype	Wildtype	Heterozygote	Wildtype	Heterozygote
Number	218	127	189	136
Percent	32%	19%	28%	20%

Table S5. Frequency of sex and genotype from wild type x heterozygote matings.

Genotype	Wildtypes		Heterozygotes		Homozygotes	
**Phenotype**	Number of embryos	**Percent**	**Number of embryos**	**Percent**	**Number of embryos**	**Percent**
**Exencephaly**					12	35.3
**Spina bifida**			1	1.4	3	8.8
**Eye defect**					3	8.8
**Coloboma**			1	1.4	10	29.4
**Eye missing**			2	2.7		
**Oedema**	2	4.4	11	14.9	19	55.9
**Bloody gut sac**	1	2.2	1	1.4		
**Midline defect**			3	4.1	1	2.9
**Smaller**	1	2.2	1	1.4	1	2.9
**Growth retarded**	3	6.7	2	2.7	2	5.9
**Webbed feet**	1	2.2			1	2.9
**Kinky tail**			1	1.4		
**Dark yolk sac**	1	2.2	1	1.4		
**Spine wrong shape**	1	2.2				
**Odd snout shape**					1	2.9
**NORMAL APPEARANCE**	35	77.8	57	77.0	8	23.5
**TOTALS**	45		74		34	

Table S6. Gross external phenotypes observed in E14.5 embryos

Supplemental Methods

Subjects. Individuals with pancreatic agenesis (defined as neonatal diabetes requiring insulin, and exocrine pancreatic insufficiency requiring oral enzyme replacement) were recruited by their clinicians for molecular genetic analysis in the Exeter Molecular Genetics Laboratory. The study was conducted in accordance with the Declaration of Helsinki, and all subjects or their parents gave informed consent for genetic testing. REC (Research Ethic Committee) reference: 17/WA/0327.

Exome sequencing. Exonic sequences were enriched from genomic DNA using Agilent's SureSelect Human All Exon kit (version 4) and then sequenced on an Illumina HiSeq 2000 sequencer using 100-bp paired-end reads. We used BWA (v0.6.2)5 to align sequence reads to the hg19 reference genome and GATK (v2.2-10) to call SNVs and indels.

Sanger sequencing confirmation. We amplified *CNOT1* exon 14 (NM_016284) using in-house designed primers (F – TGGCTTTCCCATAAAGAACG, R – TTCACCATGTTTTGGTCAGG). PCR products were sequenced on an ABI3730 capillary machine (Applied Biosystems) and analysed using Mutation Surveyor v3.98 (SoftGenetics). The bioinformatics tools SIFT, PolyPhen-2 and Align GVGD were accessed through the Alamut Visual software (Interactive Biosoftware) to predict the effect of the p.(Arg535Cys) variant *in silico*.

Mouse husbandry

All experiments were carried out in accordance with UK Home Office regulations, UK Animals (Scientific Procedures) Act 1986 and with approval from Wellcome Sanger Institute's Animal Welfare Committee. All mice were maintained in specific pathogen free facilities in individually ventilated cages at standard temperature (19-23°C) and humidity (55% ±10%), on a 12h dark, 12h light cycle. Mice were fed breeder's chow (LabDiets 5021, LabDiet, London, UK).

***Cnot1**$^{p.(Arg535Cys)}$* **mouse generation**

A one-step CRISPR/Cas9 homology-directed repair approach was used to generate *Cnot1*$^{p.(Arg535Cys)}$-mutant mice. C57BL/6N mouse zygotes were injected with *in vitro* transcribed Cas9 mRNA, single guide RNA targeting exon 14 of the *Cnot1* gene and a 126 bp single-stranded oligonucleotide (Ultramer DNA oligos, Integrated DNA Technologies) complementary to the Cas9 cleavage site. The oligonucleotide was designed to encode for the p.(ARG535CYS) substitution in *Cnot1* and harboured two additional silent mutations to limit DNA target recleavage by Cas9 after homology-directed repair. The embryos were transferred into the oviduct of pseudopregnant females the same day of microinjection. Correctly targeted, G0 mosaic offspring were identified by DNA sequencing of PCR products generated using primers CTGTAATTAATTGGTGCTTGAGCCTGACTG and CCATGTCTAAGTGCCTGATTCTGAATGCC which amplify *Cnot1* exon 14. G0 founder mice were mated to C57BL/6N mice to establish G1s for further breeding to C57BL/6N mice.

Cnot1 sgRNA ATTATAAGTTGGCGAATTGA

Cnot1$^{p.(Arg535Cys)}$-repair oligonucleotide (nucleotide substitutions in lower case)
TGGCTAACATGGTTCCTGTTTAATGTTCCCTCCTCTTTTCTTAGGGGCAGTCTCCATCgATctGCCAACTTATAAT
GCATGCAATGGCAGAATGGTACATGAGAGGGGAGCAGTATGATCAGGCCA

Genotyping

Genotyping was performed on DNA extracted from ear biopsies (adults) or yolk sac (embryos). DNA was extracted by HotSHOT[1] and genotyping performed using the LGC KASP™ system (LGC,

Teddington, UK)[2]. Plates were read using a PHERAstar plus plate reader (BMG LABTECH, Offenburg, Germany) and genotypes determined using the software KlusterCaller (LGC).

Embryo collection

For gross morphology imaging, embryos were harvested at E14.5 and dissected in cold pH7.4 PBS. They were scored for gross abnormalities and imaged using a Leica M205C microscope, DFC495 camera and LAS v4 software.

HREM

For HREM, embryos were harvested at E14.5 and dissected in 37°C HBSS (no calcium, magnesium or phenol red, LifeTech) supplemented with EDTA (0.01M LifeTech). Embryos were exsanguinated by severing of the umbilical cord and rocking in warm buffer for approximately 20 minutes, before fixation in Bouin's solution for 24 h. Embryos were stored in PBS supplemented with 0.01% sodium azide between fixation and HREM processing.

Embryos were dehydrated in methanol and embedded in methacrylate resin (JB-4, PolySciences) containing eosin B and acridine orange. Digital volume data generation was performed using high-resolution episcopic microscopy (HREM), as previously described[3-6]. Phenotype scoring was performed using virtual 2D sections, with abnormalities classified using the Mammalian Phenotype (MP) ontology[7]. Developmental staging was determined from external morphology using SD volume rendered models as described by Greyer *et al*[8].

In the original axial HREM sections of 5 *Cnot1*$^{p.(Arg535Cys)}$ homozygous mutants (two of Geyer stage (GS) 22, two of GS22+, one of GS23-), 5 CNOT *Cnot1*$^{p.(Arg535Cys)}$ heterozygous mutants (one of GS22, four of GS22+) and 18 controls (six of each, GS22, GS22+, GS23-) the ductal tissues of the pancreas were segmented employing the software package Amira 5.4.5 (Thermo Fisher Sientific) and its interactive thresholding and manual tracing tools. Separate 3D surface models of the ventral and dorsal pancreas were created and the volumes of the 3D models were determined using the "MaterialStatistics" tool of Amira. The results were statistically analysed using Excel for Mac 2011.

RNA extraction & qPCR

For pancreas dissection, embryos were harvested at E14.5 and dissected in cold pH7.4 PBS. The pancreas was removed and placed in RNAlater at 4°C for 24 h, before storage at -20°C. Tissue was homogenised in Trizol using a Qiagen TissueLyser, and RNA extracted using chloroform followed by the Qiagen MinElute kit (Qiagen, Hilden, Germany). RNA concentration and purity was evaluated by NanoDrop (Thermo Scientific, Wilmington, DE, USA). RNA integrity was further assessed using an Agilent Bioanalyzer (Agilent Technologies, Santa Clara, CA, USA). cDNA synthesis was performed using 200 ng RNA, random primers and Superscript II reverse transcriptase (Life Technologies, Carlsbad, CA, USA). qPCR was performed using Sybr Green (Applied Biosystems, Foster City, CA, USA) and run on an AB7500 qPCR machine (Applied Biosystems). The primers used were as follows:

**Gene**	**Forward Primer**	**Reverse Primer**
18S	GTAACCCGTTGAACCCCATT	CCATCCAATCGGTAGTAGCG
Gapdh	TGGTTCACACCCATCACAAACA	GGTGAAGGTCGGTGTGAACGG
Hnf1b	CAAGATGTCAGGAGTGCGCTA	CTCCCGACACTGTGATCTGC
Insulin	TGGCTTCTTCTACACACCCAAG	ACAATGCCACGCTTCTGCC
Pdx1	CAGTGGGCAGGAGGTGCTTA	CCAGATTTTGATGTGTCTCTCGG
Prss1 (Typsin-1)	GCTGACTGTGAGGCTTCCTA	AGAGTCACCCTGGCAGGAAT

Ptf1a	CATCGAGGCACCCGTTCA	GTCCAGGAAAGAGAGTGCCC
Rpl32	GGCCAGATCCTGATGCCCAAC	CAGCTGTGCTGCTCTTTCTAC
Shh	TTCCCAACGTAGCCGAGAAG	TTCCCAACGTAGCCGAGAAG

Relative expression was calculated using the ΔΔCt method, relative to the cubic mean of three reference genes (*18S*, *Gapdh* and *Rpl32*)[9].

Statistics

Genotype frequency data were analysed using Chi-Squared. All other data analysis was performed in R.

Embryo phenotype data were analysed using the Fisher's exact test assuming an additive model. Pancreas size and qPCR data were analysed using ANOVA with TukeyHSD post hoc test, including stage number as a co-variate when analysing pancreas size.

Data availability

All HREM data from this study will be made available on the DMDD website (https://dmdd.org.uk).

Supplemental References

1. Truett, G.E. *et al.* Preparation of PCR-quality mouse genomic DNA with hot sodium hydroxide and tris (HotSHOT). *Biotechniques* **29**, 52, 54 (2000).
2. Cuppen, E. Genotyping by Allele-Specific Amplification (KASPar). *CSH Protoc* **2007**, pdb.prot4841 (2007).
3. Mohun, T.J. & Weninger, W.J. Imaging heart development using high-resolution episcopic microscopy. *Curr Opin Genet Dev* **21**, 573-8 (2011).
4. Mohun, T.J. & Weninger, W.J. Embedding embryos for high-resolution episcopic microscopy (HREM). *Cold Spring Harb Protoc* **2012**, 678-80 (2012).
5. Weninger, W.J. *et al.* High-resolution episcopic microscopy: a rapid technique for high detailed 3D analysis of gene activity in the context of tissue architecture and morphology. *Anat Embryol (Berl)* **211**, 213-21 (2006).
6. Mohun, T.J. & Weninger, W.J. Generation of volume data by episcopic three-dimensional imaging of embryos. *Cold Spring Harb Protoc* **2012**, 681-2 (2012).
7. Weninger, W.J. *et al.* Phenotyping structural abnormalities in mouse embryos using high-resolution episcopic microscopy. *Dis Model Mech* **7**, 1143-52 (2014).
8. Geyer, S.H. *et al.* A staging system for correct phenotype interpretation of mouse embryos harvested on embryonic day 14 (E14.5). *J Anat* **230**, 710-719 (2017).
9. Livak, K.J. & Schmittgen, T.D. Analysis of relative gene expression data using real-time quantitative PCR and the 2(-Delta Delta C(T)) Method. *Methods* **25**, 402-8 (2001).
10. Shaw-Smith, C. *et al.* GATA4 mutations are a cause of neonatal and childhood-onset diabetes. *Diabetes* **63**, 2888-94 (2014).
11. Allen, H.L. *et al.* GATA6 haploinsufficiency causes pancreatic agenesis in humans. *Nat Genet* **44**, 20-22 (2011).
12. McMillan, T., Girgis, R. & Sellers, E.A. Neonatal diabetes and protein losing enteropathy: a case report. *BMC Med Genet* **17**, 32 (2016).
13. De Franco, E. *et al.* GATA6 mutations cause a broad phenotypic spectrum of diabetes from pancreatic agenesis to adult-onset diabetes without exocrine insufficiency. *Diabetes* **62**, 993-7 (2013).
14. Savova, R. *et al.* Marked intrafamilial variability of exocrine and endocrine pancreatic phenotypes due to a splice site mutation in GATA6. *Biotechnology & Biotechnological Equipment* **32**, 124-129 (2018).
15. Ellard, S. *et al.* Improved genetic testing for monogenic diabetes using targeted next-generation sequencing. *Diabetologia* **56**, 1958-63 (2013).
16. Catli, G. *et al.* A novel GATA6 mutation leading to congenital heart defects and permanent neonatal diabetes: a case report. *Diabetes Metab* **39**, 370-4 (2013).
17. De Franco, E. *et al.* Biallelic PDX1 (insulin promoter factor 1) mutations causing neonatal diabetes without exocrine pancreatic insufficiency. *Diabet Med* **30**, e197-200 (2013).
18. Flanagan, S.E. *et al.* Analysis of transcription factors key for mouse pancreatic development establishes NKX2-2 and MNX1 mutations as causes of neonatal diabetes in man. *Cell Metab* **19**, 146-54 (2014).
19. Gabbay, M., Ellard, S., De Franco, E. & Moises, R.S. Pancreatic Agenesis due to Compound Heterozygosity for a Novel Enhancer and Truncating Mutation in the PTF1A Gene. *J Clin Res Pediatr Endocrinol* **9**, 274-277 (2017).
20. Houghton, J.A. *et al.* Isolated Pancreatic Aplasia Due to a Hypomorphic PTF1A Mutation. *Diabetes* **65**, 2810-5 (2016).
21. Weedon, M.N. *et al.* Recessive mutations in a distal PTF1A enhancer cause isolated pancreatic agenesis. *Nat Genet* **46**, 61-64 (2014).
22. Evliyaoglu, O. *et al.* Neonatal Diabetes: Two Cases with Isolated Pancreas Agenesis due to Homozygous PTF1A Enhancer Mutations and One with Developmental Delay, Epilepsy, and

Neonatal Diabetes Syndrome due to KCNJ11 Mutation. *J Clin Res Pediatr Endocrinol* **10**, 168-174 (2018).
23. Zegre Amorim, M. *et al.* Mitchell-Riley Syndrome: A Novel Mutation in RFX6 Gene. *Case Rep Genet* **2015**, 937201 (2015).
24. Hilbrands, R. *et al.* Pancreas and gallbladder agenesis in a newborn with semilobar holoprosencephaly, a case report. *BMC Med Genet* **18**, 57 (2017).
