# Supplementary material for: A Specific CNOT1 Mutation Results in a Novel Syndrome of Pancreatic Agenesis and Holoprosencephaly through Impaired Pancreatic and Neurological Development
Source: Am J Hum Genet. 2019 Apr 18;104(5):985–9. doi: 10.1016/j.ajhg.2019.03.018 (PMC6506862; doi:10.1016/j.ajhg.2019.03.018)
Supplement: Document S2. Article plus Supplemental Data [file mmc2.pdf]

A Specific *CNOT1* Mutation Results in a Novel Syndrome of Pancreatic Agenesis and Holoprosencephaly through Impaired Pancreatic and Neurological Development

Elisa De Franco,[1,11] Rachel A. Watson,[2,11] Wolfgang J. Weninger,[3] Chi C. Wong,[2] Sarah E. Flanagan,[1] Richard Caswell,[1] Angela Green,[2] Catherine Tudor,[2] Christopher J. Lelliott,[2] Stefan H. Geyer,[3] Barbara Maurer-Gesek,[3] Lukas F. Reissig,[3] Hana Lango Allen,[1] Almuth Caliebe,[4] Reiner Siebert,[4,5] Paul Martin Holterhus,[6] Asma Deeb,[7] Fabrice Prin,[8] Robert Hilbrands,[9,10] Harry Heimberg,[9] Sian Ellard,[1] Andrew T. Hattersley,[1,12,*] and Inês Barroso[2,12,13,*]

We report a recurrent *CNOT1 de novo* missense mutation, GenBank: NM_016284.4; c.1603C>T (p.Arg535Cys), resulting in a syndrome of pancreatic agenesis and abnormal forebrain development in three individuals and a similar phenotype in mice. CNOT1 is a transcriptional repressor that has been suggested as being critical for maintaining embryonic stem cells in a pluripotent state. These findings suggest that CNOT1 plays a critical role in pancreatic and neurological development and describe a novel genetic syndrome of pancreatic agenesis and holoprosencephaly.

Discovering genes with mutations causal of pancreatic agenesis is crucial to identifying factors needed for pancreatic development. To date, pathogenic variants in six genes (*PTF1A* [MIM: 615935], *PDX1* [MIM: 260370], *GATA6* [MIM: 600001], *GATA4* [MIM: 600576], *HNF1B* [MIM: 137920], and *RFX6* [MIM: 615710]) have been reported to severely affect pancreatic development and result in pancreatic agenesis.[1] Gene discovery in pancreatic agenesis has shown both similarities and marked differences between pancreatic development in human and mouse. In both species, complete loss of function of *PTF1A*, *PDX1*, or *RFX6* results in pancreatic agenesis. In contrast, while haploinsufficiency of *GATA6* is a common cause of pancreatic agenesis in humans,[2] in mice *Gata6* knockout does not result in abnormal pancreatic development.[3] Knowledge of human pancreatic development is essential to guide progress of beta-cell replacement therapy for people with type 1 diabetes.

We investigated an international cohort of 107 individuals diagnosed with pancreatic agenesis—defined by requiring both endocrine (insulin) and exocrine (pancreatic enzymes) replacement therapy within the first 6 months of life—and identified a mutation in a known gene in 98 of them (Table S1). To identify *de novo* mutations in the remaining nine subjects, exome sequencing was performed for the probands and both their unaffected parents when available (n = 7) (Supplemental Subjects and Methods).

We identified a heterozygous missense mutation in *CNOT1* (MIM: 604917; GenBank: NM_016284.4; c.1603C>T [p.Arg535Cys]) in three individuals with pancreatic agenesis. The variant had arisen *de novo* in two of them and was not present in the DNA sample from the 3rd individual's father (maternal sample was not available for testing) (Figure 1A, Tables S2 and S3). We confirmed these results by Sanger sequencing (Supplemental Subjects and Methods, Figure S1). The p.Arg535Cys variant is absent from dbSNP138, DECIPHER, and GnomAD and affects a residue which is highly conserved across species (up to *C. elegans*) (Figure 1B). All three *in silico* prediction tools used (AlignGVGD, PolyPhen2, and SIFT accessed through AlamutVisual) predicted the variant to have a deleterious effect on protein function (Supplemental Subjects and Methods).

The three individuals who were heterozygous for the *CNOT1* p.Arg535Cys variant had strikingly similar clinical features (see Supplemental Note). In addition to pancreatic agenesis, all three had definite (n = 2) or possible holoprosencephaly (Figure 1A, Table S4), a disorder in which the prosencephalon (forebrain of the embryo) fails to develop into two hemispheres. P01 and P02 (who was previously reported by Hilbrands et al.[4]) both had partial/semi-lobar holoprosencephaly, while P03 has dysmorphic features which could be consistent with holoprosencephaly (prominent central incisors and occiput, highly arched palate, and low-set ears) but brain MRI was declined by his parents

[1]Institute of Biomedical and Clinical Science, University of Exeter Medical School, EX2 5DW Exeter, UK; [2]Wellcome Sanger Institute, CB10 1SA Hinxton, UK; [3]Centre for Anatomy and Cell Biology & MIC, Medical University of Vienna, 1090 Vienna, Austria; [4]Institute of Human Genetics, Christian-Albrechts-University 24105 Kiel and University Hospital Schleswig-Holstein, 24105 Kiel, Germany; [5]Institute of Human Genetics, Ulm University & Ulm University Medical Center, 89081 Ulm, Germany; [6]Department of Pediatrics, Division of Pediatric Endocrinology and Diabetes, Christian-Albrechts-University 24105 Kiel and University Hospital Schleswig-Holstein, 24105 Kiel, Germany; [7]Paediatric Endocrinology Department, Mafraq Hospital, 2951 Abu Dhabi, United Arab Emirates; [8]The Francis Crick Institute, NW1 1ST London, UK; [9]Vrije Universiteit Brussel, 1090 Brussels, Belgium; [10]Universitair Ziekenhuis Brussel, 1090 Brussels, Belgium
[11]These authors contributed equally to this work
[12]These authors contributed equally to this work
[13]Present address: MRC Epidemiology Unit, Institute of Metabolic Science, University of Cambridge, CB2 0SL Cambridge, UK
*Correspondence: a.t.hattersley@exeter.ac.uk (A.T.H.), ines.barroso@mrc-epid.cam.ac.uk (I.B.)
https://doi.org/10.1016/j.ajhg.2019.03.018.
© 2019 The Authors. This is an open access article under the CC BY license (http://creativecommons.org/licenses/by/4.0/).

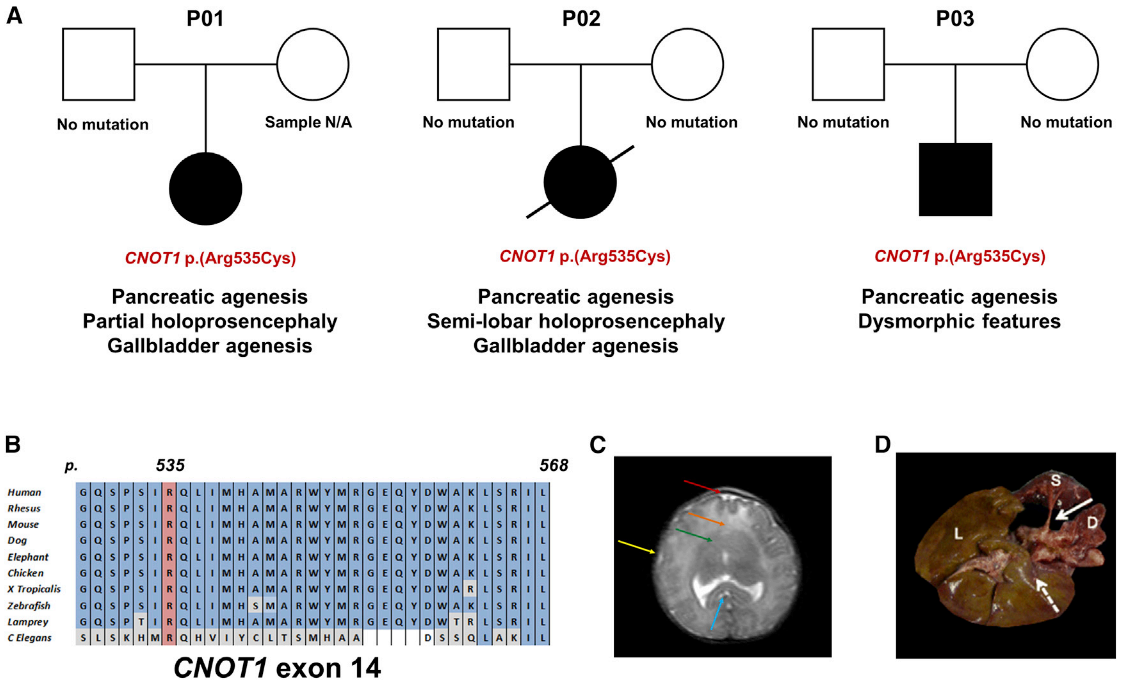

Figure 1. Genetic and Clinical Findings in Individuals with Pancreatic Agenesis
(A) Partial pedigrees and clinical features of the three individuals with the heterozygous *CNOT1* p.Arg535Cys mutation.
(B) Conservation of CNOT1 residues 529 to 568 across 10 representative species. Residue p.Arg535 is highlighted in red. Residues identical to the human CNOT1 protein are highlighted in blue, differences are highlighted in gray.
(C) Coronal brain MRI of P02 showing absence of the anterior interhemispheric fissure (red arrow), fusion of the frontal lobes (orange arrow), absence of frontal horns (green arrow), absence of the sylvian fissures (yellow arrow). Splenium of the corpus callosum is visible (blue arrow).
(D) Post mortem image of P02's liver (L), spleen (S), and duodenum (D). White arrow shows the orthotopic location of the pancreas, which is absent. Dashed arrow indicated site in which the absent gallbladder would be expected to be.

and the diagnosis could not therefore be confirmed. All three individuals had very low birth weight (Z-score < -2), likely due to insulin deficiency in the last trimester of pregnancy, when insulin is the main fetal growth factor. Consistent with insulin deficiency *in utero*, the three case subjects all developed diabetes very early (2/3 diagnosed at 1 day and 1 at 13 weeks). P01 and P02 also had gallbladder agenesis, a clinical feature frequently associated with pancreatic agenesis.

The DDD study[5] has identified *de novo CNOT1* variants in three individuals with developmental delay (two missense—p.Leu2323Phe and p.Arg623Trp—and a nonsense—p.Gln33*—variant) but none of them had holoprosencephaly or diabetes. Since our three case subjects were all heterozygous for the same novel missense *CNOT1* variant and none of the DDD participants with heterozygous *de novo CNOT1* variants had pancreatic or neurological structural malformations, we hypothesized that a mutation-specific mechanism rather than loss of function was responsible for the phenotype seen in our case subjects. We therefore generated a mouse line harboring the *Cnot1*$^{\text{p.(Arg535Cys)}}$ mutation using CRISPR (Supplemental Subjects and Methods).

Heterozygous mice were born at a lower than expected frequency (Table S5), but without an obvious phenotype, while homozygosity for the mutation was embryonically lethal. At E14.5, embryos were still alive and present at expected Mendelian ratios and were therefore collected to assess their phenotype (Supplemental Subjects and Methods). Upon dissection, several gross morphological abnormalities were apparent in homozygotes, notably exencephaly, eye defects (mostly coloboma), and edema (Figures 2A, 2B, and S2; Table S6).

High-resolution episcopic microscopy (HREM) highlighted a significant reduction in the size of the pancreas in homozygous embryos in addition to several other abnormalities (Figures 2C–2G, Supplemental Subjects and Methods, DMDD website). The reduction in pancreatic size was found to be predominantly due to a smaller dorsal pancreas (Figures 2H and S3). These results provide compelling evidence for the role of *Cnot1*$^{\text{p.(Arg535Cys)}}$ in pancreatic development.

Expression analysis of pancreatic developmental factors on RNA extracted from pancreatic tissue in E14.5 wild-type, *Cnot1*$^{\text{p.(Arg535Cys)/p.(Arg535Cys)}}$, and *Cnot1*$^{\text{p.(Arg535Cys)}}$ embryos showed a significant increase of *Shh* expression in homozygous embryos, with decreased expression in *Pdx1*, *Ins*, *Hnf1b*, and *Ptf1a* (Figure 3A). No difference in expression was detected for *Gata6* (Figure 3A) and *Rxra* (Figure S4).

The pancreatic and neurological phenotypes observed in *Cnot1*$^{\text{p.(Arg535Cys)/p.(Arg535Cys)}}$ E14.5 mouse embryos are consistent with the pancreatic agenesis and holoprosencephaly observed in the three case subjects, confirming that the *de novo CNOT1* mutation is indeed the cause of their

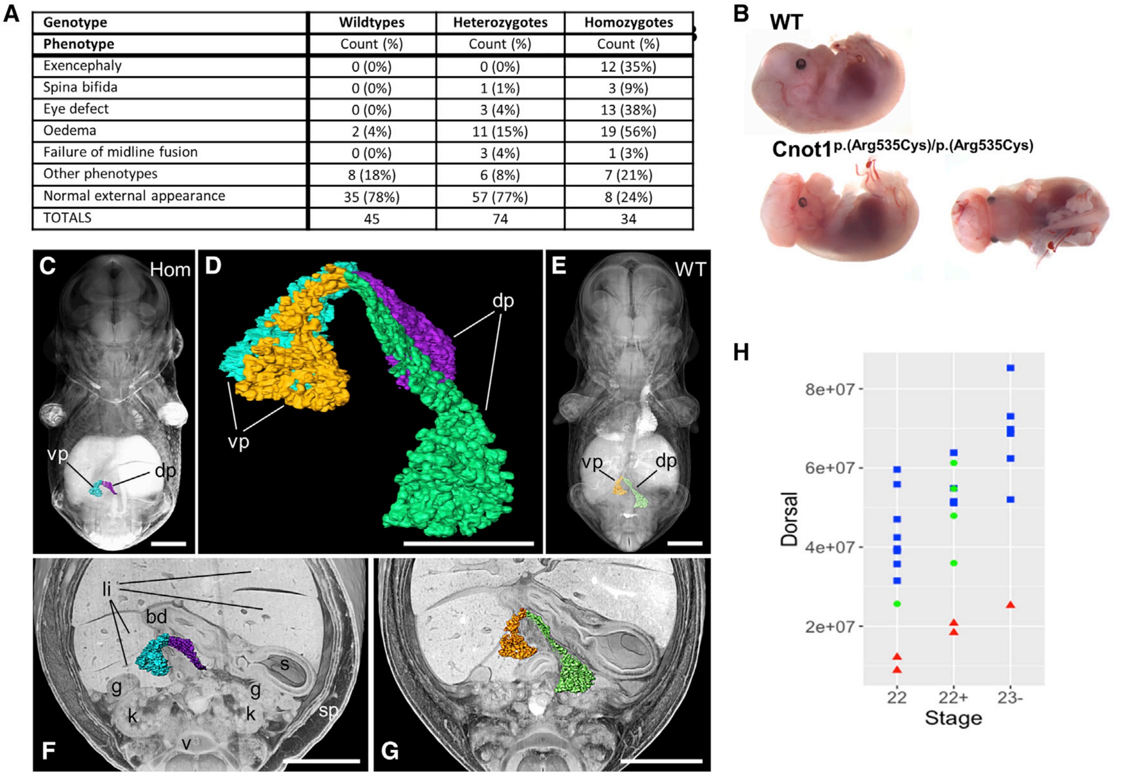

Genotype	Wildtypes	Heterozygotes	Homozygotes
Phenotype	Count (%)	Count (%)	Count (%)
Exencephaly	0 (0%)	0 (0%)	12 (35%)
Spina bifida	0 (0%)	1 (1%)	3 (9%)
Eye defect	0 (0%)	3 (4%)	13 (38%)
Oedema	2 (4%)	11 (15%)	19 (56%)
Failure of midline fusion	0 (0%)	3 (4%)	1 (3%)
Other phenotypes	8 (18%)	6 (8%)	7 (21%)
Normal external appearance	35 (78%)	57 (77%)	8 (24%)
TOTALS	45	74	34

Figure 2. Neurological and Pancreatic Abnormalities in Mouse Embryos Homozygous for the *Cnot1* p.Arg535Cys Mutation
(A) Table listing the gross external phenotypes observed in E14.5 embryos. Numbers do not add to total as many embryos displayed multiple phenotypes. Significance by Fisher's exact test, assuming an additive model. Exencephaly, $p = 3.2 \times 10^{-9}$; spina bifida, $p = 0.027$; eye defect, $p = 5.5 \times 10^{-8}$; edema, $p = 2.6 \times 10^{-7}$; midline defect, ns.
(B) Images showing representative E14.5 embryos: top shows wild-type embryo, bottom shows embryo homozygous for the *CNOT1* p.Arg535Cys mutation with exencephaly and coloboma.
(C and E) Coronally sectioned, semi-transparent 3D volume models of stage-matched E14.5 embryos with superimposed models of the pancreas of homozygous (C) and wild-type (E) embryos.
(D) Overlay of extracted surface models of the pancreas of homozygous (blue, magenta) and wild-type embryos (orange, green).
(F and G) Coronally sectioned solid 3D volume rendered model of the abdomen of the embryos shown in (C) and (E) with superimposed pancreas. dp, dorsal pancreas; vp, ventral pancreas; li, liver lobes; s, stomach; sp, spleen; k, kidney; g, gonad; bd, bile duct.
Scale bars: 1,000 μm in (C), (E)–(G); 500 μm in (D).
(H) Graph showing the volume of the dorsal pancreas of E14.5 embryos in μm^3. Blue squares show wild types, green circles are heterozygotes, and red triangles are homozygotes. Data analyzed using ANOVA with TukeyHSD posthoc test, effect of genotype $p = 8.85 \times 10^{-8}$; post hoc WT-Hom, $p < 10^{-10}$; Het-Hom, $p = 1.36 \times 10^{-4}$, WT-Het, ns.

disease. Mice required a homozygous mutation in *Cnot1* to display a pancreatic and brain phenotype while a heterozygous *CNOT1* mutation resulted in the phenotype in three individuals in our cohort. This has been described with other pancreatic developmental genes (e.g., *HNF1B*) and supports the hypothesis that the early stages of pancreatic development are not identical in mice and humans.[6]

The CNOT1 protein has not previously been suggested to have a role in pancreatic development; it is known to act both as scaffold of the CCR4-NOT complex and as an independent factor. As such, it mediates transcriptional repression[7] and is expressed extremely early during embryonic development (E3.5 in the inner cell mass in mice[8]). *In vitro* studies have proposed that CNOT1 plays a critical role in maintaining human and mice embryonic stem cells in a pluripotent state by inhibiting primitive endoderm factors.[8] *CNOT1* expression peaks in undifferentiated human induced pluripotent stem (iPS) cells compared to subsequent stages of *in vitro* differentiation toward pancreatic endocrine cells,[9] supporting its fundamental role in stem cells.

The increased expression of Shh in pancreatic tissue extracted from Cnot1$^{p.(Arg535Cys)/p.(Arg535Cys)}$ embryos would be consistent with a model in which the *CNOT1* p.Arg535Cys mutation results in embryonic stem cells being maintained in an undifferentiated state through SHH-mediated inhibition of differentiation. SHH is a key developmental factor that is known to be crucial for pancreatic and brain development. Heterozygous loss-of-function mutations in *SHH* cause holoprosencephaly (MIM: 142945) and studies in both mouse and human embryos have shown that *SHH* expression needs to be repressed in the dorsal foregut endoderm for successful differentiation toward dorsal pancreas.[6,10] A recent study has suggested that the transcription factors Gata4 and Gata6 (mutations in which are a

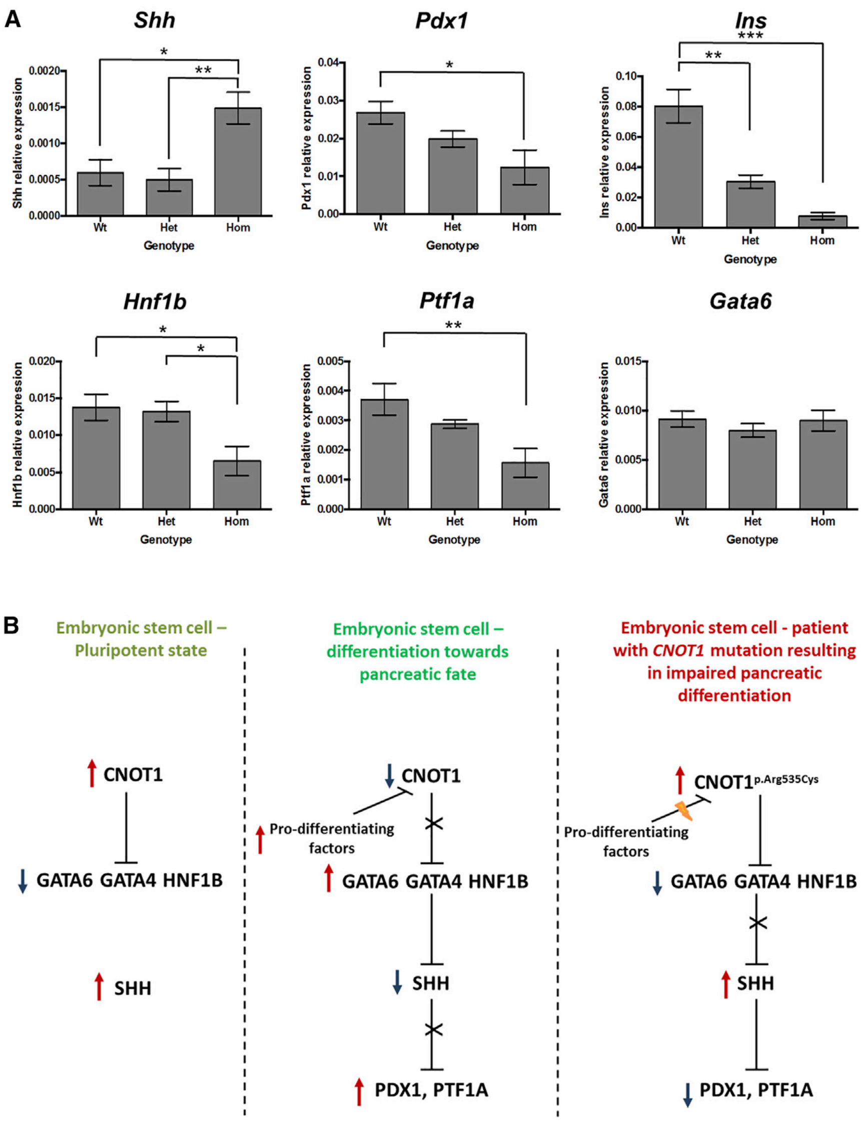

Figure 3. Expression Data and Possible Mechanism Involving CNOT1 in Pancreatic Development

(A) Graphs showing relative expression of genes in the pancreas of E14.5 embryos. Bars show mean ± SE. Data analyzed using ANOVA with TukeyHSD posthoc test. Results of posthoc tests shown on graphs, $^{*}p < 0.05$, $^{**}p < 0.01$, $^{***}p < 0.001$. *Shh*, effect of genotype $p = 0.0107$; *Pdx1*, effect of genotype $p = 0.0189$; *Ins*, effect of genotype $p = 7.03 \times 10^{-6}$; *Hnf1b*, effect of genotype $p = 0.0294$; *Ptf1a*, effect of genotype $p = 0.00781$; *Gata6*, effect of genotype p = ns. n = 4–12 animals per genotype.

(B) Schematic representation of the proposed role for CNOT1 in pancreatic development.

cause of pancreatic agenesis) regulate pancreatic endoderm identity by directly inhibiting Shh in mice.[11] It is therefore possible that the p.Arg535Cys variant results in CNOT1 maintaining its inhibition activity on the GATA and other early differentiation factors and, as a consequence, SHH expression is not repressed (Figure 3B). Increased expression of *Shh* and decreased expression of *Pdx1*, *Ins*, *Hnf1b*, and *Ptf1a* detected in RNA extracted from pancreatic tissue in the E14.5 *Cnot1*$^{\mathrm{p.(Arg535Cys)/p.(Arg535Cys)}}$ embryos would support this hypothesis. However, Gata4 expression could not be assessed as the assay specificity was too low and Gata6 expression was not found to be reduced. It is possible that Gata6 activity is actually inhibited earlier during development and then re-activated by a different pathway (Gata6 is needed for development of most endodermal-derived organs and heart) or could be inhibited by a different mechanism that does not result in reduced expression. The *CNOT1* p.Arg535Cys mutation also affects neurological development in both our case subjects and mouse embryos. It is possible that this mutation results in ectopic SHH expression during brain development. This would be consistent with previous reports of Shh ectopic expression impairing midline development.[12] Another possibility is that the effect of the *CNOT1* mutation on SHH signaling differs between the brain and the pancreas, resulting in a reduced expression in the developing brain and increased expression during pancreatic development. Further experiments, ideally on younger embryos and human iPS cells, are needed in order to elucidate the mechanism by which the *CNOT1* p.Arg535Cys mutation results in impaired pancreatic and neurological development.

Our study identifies a spontaneous *CNOT1* p.Arg535Cys mutation as the genetic cause of a rare syndrome of pancreatic agenesis and holoprosencephaly, highlighting a previously unsuspected role of CNOT1 as a key factor in both pancreatic and neurological development. This is the 7th gene causative of pancreatic agenesis described so far and the first pancreatic agenesis gene that is thought to be important for maintaining stem cells' pluripotency. These findings suggest a new mechanism by which impairment of the very early stages of development result in pancreatic agenesis and abnormal brain development.

Supplemental Data

Supplemental Data can be found online at https://doi.org/10.1016/j.ajhg.2019.03.018.

Acknowledgments

The authors thank the families for participating in this study. We are grateful to Anna-Marie Johnson and Benjamin Bunce for their technical assistance and to Dr. Jane Ferguson and Dr. Joerg Detlev Moritz for assistance with the MRI images. We are grateful to

Emma Siragher and Monika Dabrowska for assistance with embryo dissection, Antonella Galli for advice on embryo work, members of the DMDD consortium at the Francis Crick Institute for imaging the embryos, Julia Rose for assistance with HREM image analysis, members of the Research Support Facility and Mouse Pipelines at the Wellcome Sanger Institute for mouse management and husbandry, Rachel Moore and Eleanor Wheeler for help with the mouse statistical analysis, Bill Bottomley for help with mouse generation, and Elizabeth Wynn for help with genotyping. We would also like to thank Prof. Lori Sussell for useful discussion on the SHH pathway.

I.B. is funded by Wellcome (WT206194). A.T.H. and S.E. are the recipients of a Wellcome Trust Senior Investigator award (grant WT098395/Z/12/Z) and A.T.H. is employed as a core member of staff within the NIHR funded Exeter Clinical Research Facility and is an NIHR senior investigator. E.D.F. was a Naomi Berrie Fellow in Diabetes Research (grant 50565) during the study. S.E.F. has a Sir Henry Dale Fellowship jointly funded by the Wellcome Trust and the Royal Society (grant number 105636/Z/14/Z). C.C.W. holds a Wellcome Trust Intermediate Clinical Fellowship (grant number 105914/Z/14/Z). H.H. is funded by the Research Foundation-Flanders (FWO), the VUB Research Council, and Stichting Diabetes Onderzoek Nederland.

Declaration of Interests

The authors declare no competing interests.

Received: December 3, 2018
Accepted: March 18, 2019
Published: April 18, 2019

Web Resources

DECIPHER, https://decipher.sanger.ac.uk/
DMDD, https://dmdd.org.uk
GenBank, https://www.ncbi.nlm.nih.gov/genbank/
gnomAD Browser, https://gnomad.broadinstitute.org/
OMIM, http://www.omim.org/

References

1. De Franco, E., and Ellard, S. (2015). Genome, exome, and targeted next-generation sequencing in neonatal diabetes. Pediatr. Clin. North Am. *62*, 1037–1053.
2. Allen, H.L., Flanagan, S.E., Shaw-Smith, C., De Franco, E., Akerman, I., Caswell, R., Ferrer, J., Hattersley, A.T., Ellard, S.; and International Pancreatic Agenesis Consortium (2011). GATA6 haploinsufficiency causes pancreatic agenesis in humans. Nat. Genet. *44*, 20–22.
3. Morrisey, E.E., Tang, Z., Sigrist, K., Lu, M.M., Jiang, F., Ip, H.S., and Parmacek, M.S. (1998). GATA6 regulates HNF4 and is required for differentiation of visceral endoderm in the mouse embryo. Genes Dev. *12*, 3579–3590.
4. Hilbrands, R., Keymolen, K., Michotte, A., Marichal, M., Cools, F., Goossens, A., Veld, P.I., De Schepper, J., Hattersley, A., and Heimberg, H. (2017). Pancreas and gallbladder agenesis in a newborn with semilobar holoprosencephaly, a case report. BMC Med. Genet. *18*, 57.
5. Firth, H.V., Wright, C.F.; and DDD Study (2011). The Deciphering Developmental Disorders (DDD) study. Dev. Med. Child Neurol. *53*, 702–703.
6. Jennings, R.E., Berry, A.A., Kirkwood-Wilson, R., Roberts, N.A., Hearn, T., Salisbury, R.J., Blaylock, J., Piper Hanley, K., and Hanley, N.A. (2013). Development of the human pancreas from foregut to endocrine commitment. Diabetes *62*, 3514–3522.
7. Winkler, G.S., Mulder, K.W., Bardwell, V.J., Kalkhoven, E., and Timmers, H.T. (2006). Human Ccr4-Not complex is a ligand-dependent repressor of nuclear receptor-mediated transcription. EMBO J. *25*, 3089–3099.
8. Zheng, X., Dumitru, R., Lackford, B.L., Freudenberg, J.M., Singh, A.P., Archer, T.K., Jothi, R., and Hu, G. (2012). Cnot1, Cnot2, and Cnot3 maintain mouse and human ESC identity and inhibit extraembryonic differentiation. Stem Cells *30*, 910–922.
9. van de Bunt, M., Lako, M., Barrett, A., Gloyn, A.L., Hansson, M., McCarthy, M.I., Beer, N.L., and Honoré, C. (2016). Insights into islet development and biology through characterization of a human iPSC-derived endocrine pancreas model. Islets *8*, 83–95.
10. Apelqvist, A., Ahlgren, U., and Edlund, H. (1997). Sonic hedgehog directs specialised mesoderm differentiation in the intestine and pancreas. Curr. Biol. *7*, 801–804.
11. Xuan, S., and Sussel, L. (2016). GATA4 and GATA6 regulate pancreatic endoderm identity through inhibition of hedgehog signaling. Development *143*, 780–786.
12. Huang, X., Litingtung, Y., and Chiang, C. (2007). Ectopic sonic hedgehog signaling impairs telencephalic dorsal midline development: implication for human holoprosencephaly. Hum. Mol. Genet. *16*, 1454–1468.

The American Journal of Human Genetics, Volume *104*

Supplemental Data

A Specific *CNOT1* Mutation Results in a Novel Syndrome of Pancreatic Agenesis and Holoprosencephaly through Impaired Pancreatic and Neurological Development

Elisa De Franco, Rachel A. Watson, Wolfgang J. Weninger, Chi C. Wong, Sarah E. Flanagan, Richard Caswell, Angela Green, Catherine Tudor, Christopher J. Lelliott, Stefan H. Geyer, Barbara Maurer-Gesek, Lukas F. Reissig, Hana Lango Allen, Almuth Caliebe, Reiner Siebert, Paul Martin Holterhus, Asma Deeb, Fabrice Prin, Robert Hilbrands, Harry Heimberg, Sian Ellard, Andrew T. Hattersley, and Inês Barroso

Supplemental Data

Supplemental Note: Case reports

Case P01

P01 is a 13 years and 9 months-old girl born to non-consanguineous parents. She was born at 38+4 weeks gestation after a pregnancy complicated by growth retardation and corpus callosum agenesis detected sonographically. She was born small for gestational age (birth-weight 1340g, length 41 cm and OFC 30 cm). She developed insulin-dependent diabetes on day 1 of life. Abdominal ultrasound and MRI failed to detect pancreas and gallbladder, but the ductus choledochus was present and the intrahepatic gall ducts were normal. Gamma-glutamyl-transpeptidase was transiently elevated in the neonatal period (800 IU). Therapy with oral pancreatic enzymes for pancreatic exocrine insufficiency was commenced before discharge. Brain MRI confirmed a diagnosis of lobular holoprosencephaly with dysplastic frontal horns of the lateral ventricles, missing septum pellucidum, broadly joined cella media of the lateral ventricles, and hypoplasia of the corpus callosum (only present in the rostral portion of the corpus and the splenium, frontal parts not present). Muscle weakness, low-set ears and cardiac extrasystoly were also identified in the neonatal period. At the age of 3 years she developed complex focal seizures and she has been on sultiame therapy since. At the age of 3 years and one month, her height was 87cm (<3rd centile) and her weight was 11.6kg (between 3rd and 10th). At the age of 9 years she was reported to have mild learning difficulties. She is on growth hormone therapy (SGA-indication, pituitary growth hormone deficiency was excluded). Her current height is 152.2 cm (10th centile), weight 55kg (between 50th and 75th).

Case P02

P02 has been previously reported by Hilbrands *et al*[24]. Briefly, she was the second child of non-consanguineous parents. She was born at 38 weeks gestation, with a birth weight of 1100g, after an uneventful pregnancy complicated by intrauterine growth retardation. There was not family history of diabetes or brain anomalies. Dysmorphic features such as receding forehead, cylindrical nose, mild hypotelorism, dysplastic left ear, and hypoplastic zygomatic bone; and abducted thumbs were noted at birth. Diabetes was diagnosed in the first day of life and insulin treatment commenced. Exocrine insufficiency was confirmed during the second week of life and treatment with oral pancreatic enzymes was started. Complete absence of the pancreas and gallbladder were initially detected by abdomen MRI. Brain MRI confirmed semilobar holoprosencephaly with absent corpus callosum. She died at 12 weeks and 3 days.

Case P03

P03 was born after 39 weeks gestation and his birth weight was 1900g. His parents are not related and no family history of diabetes was reported. He was diagnosed with diabetes at the age of 3 weeks and treated with insulin. An abdominal ultrasound failed to detect a pancreas and a negative immune-reactive trypsin test confirmed exocrine pancreatic insufficiency, which was treated with oral pancreatic enzymes. Mild dysmorphic features (prominent occiput, low-set ears, high arched palate, prominent central incisors) and transient elevated liver enzymes (Alt 80 (NR 5-45), AST 66 (5-35), GGT 59 (3-22)) were reported. At last assessment (aged 16 years old) was still treated with oral pancreatic enzymes and insulin (dose 0.8U/kg/day on insulin pump). He was attending a normal school and there were no developmental concerns. A brain MRI scan was declined by his parents.

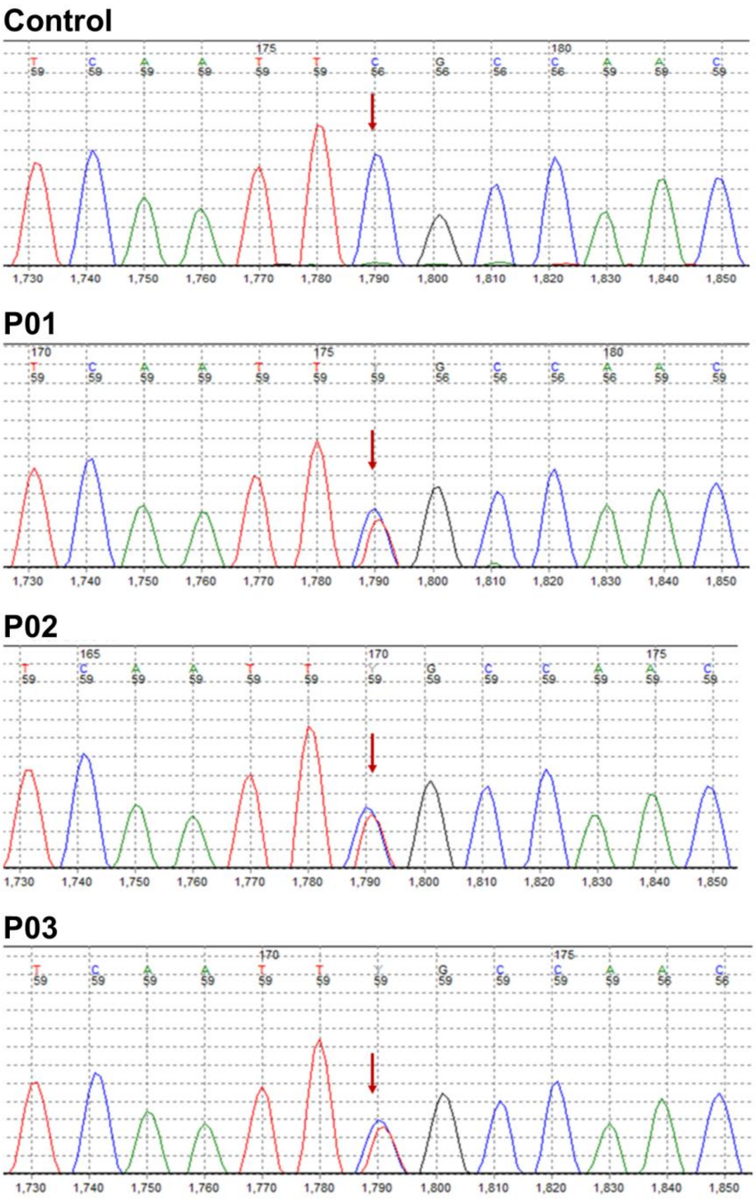

Figure S1. Sanger sequencing traces for the NM_016284.4(*CNOT1*):c.1603C>T, p.(Arg535Cys) variant (indicated by the red arrow).

HOMOZYGOTES

Normal external appearance:

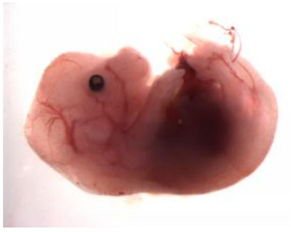

Exencephaly & Coloboma:

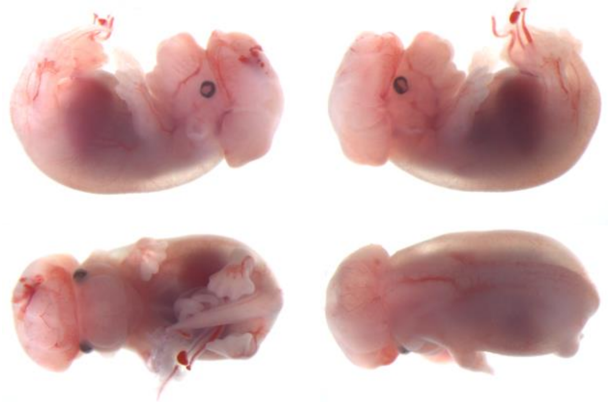

Oedema: Exencephaly & Spina

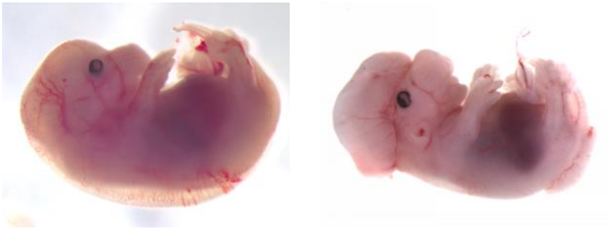

HETEROZYGOTES

Normal external appearance: Oedema:

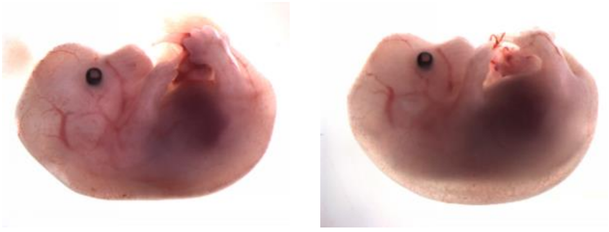

Midline defect & eye defect:

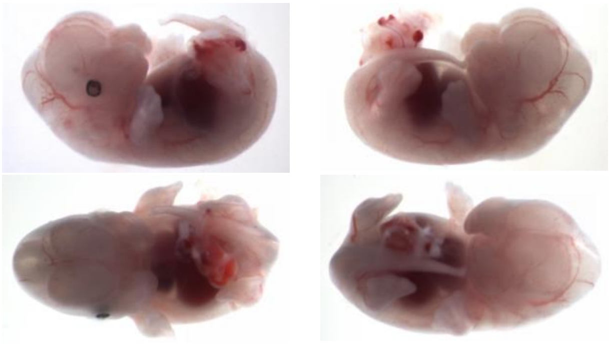

Figure S2. External morphology of E14.5 embryos.

A

Stage	Genotype	n	Dorsal pancreas volume (µm³)	Ventral pancreas volume (µm³)	Total pancreas volume (µm³)	Ratio Ventral:Dorsal
22	WT	6	39,205,654	16,695,357	55,901,011	0.42
	Het	1	25,633,746	12,693,213	38,326,959	0.50
	Hom	2	10,499,964	17,188,757	27,688,721	1.68
22+	WT	6	56,145,465	24,802,275	80,947,741	0.44
	Het	4	49,970,453	24,986,921	74,957,374	0.50
	Hom	2	19,544,099	20,346,290	39,890,389	1.05
23-	WT	6	68,546,795	32,482,914	101,029,709	0.48
	Het	0				
	Hom	1	25,231,986	30,382,804	55,614,790	1.20

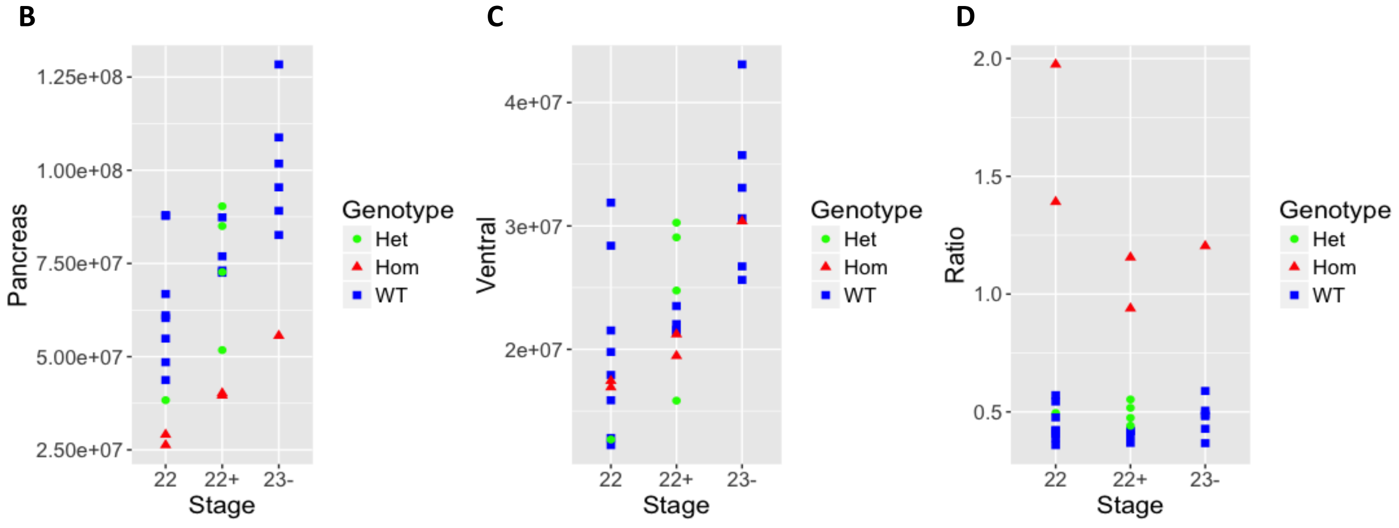

Figure S3. Pancreas size is reduced in E14.5 mutant embryos

A: Table showing the mean data for the pancreas volume determination.

B & C. Graphs showing the volume of the total (A) and ventral (B)pancreas in µm^3. Data analysed using ANOVA with TukeyHSD posthoc test. B: effect of genotype p=2.38x10^{-5}; post-hoc WT-Hom, p=1.46x10^{-5}; Het-Hom, p=0.007, WT-Het, ns. C: effect of genotype: ns.

D. Ratio of ventral:dorsal pancreas volume. Data analysed using ANOVA with TukeyHSD posthoc test; effect of genotype p=5.6x10^{-10}; post-hoc WT-Hom, p<10^{-10}; Het-Hom, p=1x10^{-7}, WT-Het, ns

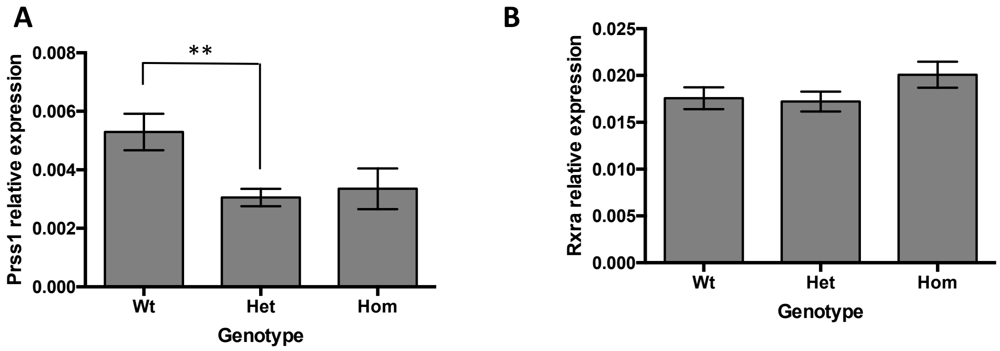

Figure S4. Relative expression of genes in the pancreas of E14.5 embryos. Bars show mean +/- SE. Data analysed using ANOVA with TukeyHSD posthoc test. Results of posthoc tests shown on graphs, * p<0.05, ** p<0.01, *** p<0.001. A: Prss1; effect of genotype p=0.00612. B. Rxra; effect of genotype ns.

ID	gene	Transcript	Nucleotide Change	Mutation Name	Exon Intron	Reference if previously reported
P01	*CNOT1*	NM_016284.4	c.1603C>T	p.(Arg535Cys)	Exon 14	
P02	*CNOT1*	NM_016284.4	c.1603C>T	p.(Arg535Cys)	Exon 14	
P03	*CNOT1*	NM_016284.4	c.1603C>T	p.(Arg535Cys)	Exon 14	
P04	*GATA4*	NM_002052.3	c.819C>A	p.(Asn273Lys)	Exon 4	[10]
P05	*GATA4*	NM_002052.3	c.(?_-554)_(1329+2_?)del	p.?	Exon 1-7	[10]
P06	*GATA4*	NM_002052.3	c.(?_-554)_(1329+2_?)del	p.?	Exon 1-7	
P07	*GATA6*	NM_005257.5	c.(?_-265)_(1135+2_?)del	p.?	Exon 1-2	
P08	*GATA6*	NM_005257.5	c.(?_-37)_(1788+2_?)del	p.?	Exon 1-7	
P09	*GATA6*	NM_005257.5	c.(?_-1)_(1788+2_?)del	p.?	Exon 1-7	
P10	*GATA6*	NM_005257.5	c.635_660del	p.(Pro212fs)	Exon 2	
P11	*GATA6*	NM_005257.5	c.701delC	p.(Pro234fs)	Exon 2	[11]
P12	*GATA6*	NM_005257.5	c.744delG	p.(Pro249fs)	Exon 2	[12]
P13	*GATA6*	NM_005257.5	c.877_880delinsTAC	p.(Val293fs)	Exon 2	
P14	*GATA6*	NM_005257.5	c.969C>A	p.(Tyr323*)	Exon 2	[13]
P15	*GATA6*	NM_005257.5	c.1013C>A	p.(Ser338*)	Exon 2	
P16	*GATA6*	NM_005257.5	c.1036_1042del	p.(Thr346fs)	Exon 2	[13]
P17	*GATA6*	NM_005257.5	c.1108_1121dup	p.(Glu375fs)	Exon 2	[11]
P18	*GATA6*	NM_005257.5	c.1136-2A>G	p.?	Intron 2	[13,14]
P19	*GATA6*	NM_005257.5	c.1242C>A	p.(Cys414*)	Exon 3	
P20	*GATA6*	NM_005257.5	c.1296del	p.(Lys432fs)	Exon 3	[15]
P21	*GATA6*	NM_005257.5	c.1303-10C>G	p.?	Intron 3	[11]
P22	*GATA6*	NM_005257.5	c.1303-2A>G	p.?	Intron 4	
P23	*GATA6*	NM_005257.5	c.1303-1G>T	p.?	Intron 4	[13]
P24	*GATA6*	NM_005257.5	c.1330T>C	p.(Cys444Arg)	Exon 4	
P25	*GATA6*	NM_005257.5	c.1354A>G	p.(Thr452Ala)	Exon 4	[11]
P26	*GATA6*	NM_005257.5	c.1366C>T	p.(Arg456Cys)	Exon 4	[11]
P27	*GATA6*	NM_005257.5	c.1366C>T	p.(Arg456Cys)	Exon 4	
P28	*GATA6*	NM_005257.5	c.1367G>A	p.(Arg456His)	Exon 4	[11]
P29	*GATA6*	NM_005257.5	c.1369A>G	p.(Arg457Gly)	Exon 4	
P30	*GATA6*	NM_005257.5	c.1396A>G	p.(Asn466Asp)	Exon 4	
P31	*GATA6*	NM_005257.5	c.1397A>G	p.(Asn466Ser)	Exon 4	[15,16]
P32	*GATA6*	NM_005257.5	c.1399G>A	p.(Ala467Thr)	Exon 4	[11]
P33	*GATA6*	NM_005257.5	c.1406G>A	p.(Gly469Glu)	Exon 4	[13]
P34	*GATA6*	NM_005257.5	c.1417A>C	p.(Lys473Gln)	Exon 4	[11]
P35	*GATA6*	NM_005257.5	c.1429-41_1441del	p.?	Intron 4	[13]
P36	*GATA6*	NM_005257.5	c.1429-8T>G	p.?	Intron 4	[13]
P37	*GATA6*	NM_005257.5	c.1435A>G	p.(Arg479Gly)	Exon 5	[13]
P38	*GATA6*	NM_005257.5	c.1448_1455del	p.(Met483fs)	Exon 5	[11,15]
P39	*GATA6*	NM_005257.5	c.1498_1501del	p.(Lys500fs)	Exon 5	[11]
P40	*GATA6*	NM_005257.5	c.1516+1G>C	p.?	Intron 5	[11]
P41	*GATA6*	NM_005257.5	c.1516+4A>G	p.?	Intron 5	[11]
P42	*PDX1*	NM_000209.3	c.455C>G/c.455C>G	p.(Ala152Gly)/p.(Ala152Gly)	Exon 2	[17,18]
P43	*PDX1*	NM_000209.3	c.478C>A/c.478C>A	p.(Glu160Lys)/p.(Glu160Lys)	Exon 2	
P44	*PDX1*	NM_000209.3	c.488A>G/c.488A>G	p.(Lys163Arg)/p.(Lys163Arg)	Exon 2	
P45	*PDX1*	NM_000209.3	c.518G>C/c.518G>C	p.(Arg173Pro)/p.(Arg173Pro)	Exon 2	
P46	*PDX1*	NM_000209.3	c.524G>T/c.524G>T	p.(Arg175Leu)/p.(Arg175Leu)	Exon 2	
P47	*PTF1A*	NM_178161.2	c.1A>G/c.1A>G	p.(Met1?)/p.(Met1?)	Exon 1	
P48	*PTF1A*	NM_178161.2	c.399dup/c.399dup	p.(Pro134fs)/p.(Pro134fs)	Exon 1	
P49	*PTF1A*	NM_178161.2	c.437_462del/g.23508442A>G	p.(Ala146fs)/p.?	Exon 1/Enhancer	[19]
P50	*PTF1A*	NM_178161.2	c.571C>A/c.571C>A	p.(Pro191Thr)p.(Pro191Thr)	Exon 1	[20]

P51	*PTF1A*	NM_178161.2	c.571C>A/c.571C>A	p.(Pro191Thr)p.(Pro191Thr)	Exon 1	[20]
P52	*PTF1A*	NM_178161.2	c.571C>A/c.571C>A	p.(Pro191Thr)p.(Pro191Thr)	Exon 1	
P53	*PTF1A*	NM_178161.2	c.571C>A/c.571C>A	p.(Pro191Thr)p.(Pro191Thr)	Exon 1	
P54	*PTF1A*	NM_178161.2	c.571C>A/c.571C>A	p.(Pro191Thr)p.(Pro191Thr)	Exon 1	
P55	*PTF1A*	NM_178161.2	c.784+4A>G/c.784+4A>G	p.?/p.?	Intron 1	[18]
P56	*PTF1A*	NM_178161.2	c.886C>T/c.886C>T	p.(Arg296*)/p.(Arg296*)	Exon 2	
P57	*PTF1A*		g.23508124-?_23508633+?del/g.23508124-?_23508633+?	p.?/p.?	Enchancer	[21]
P58	*PTF1A*		g.23508124-?_23508633+?del/g.23508124-?_23508633+?	p.?/p.?	Enchancer	
P59	*PTF1A*		g.23508124-?_23508633+?del/g.23508124-?_23508633+?	p.?/p.?	Enchancer	
P60	*PTF1A*		g.23508305A>G/g.23508305A>G	p.?/p.?	Enchancer	[21]
P61	*PTF1A*		g.23508336G>T/g.23508336G>T	p.?/p.?	Enchancer	
P62	*PTF1A*		g.23508363A>G/g.23508363A>G	p.?/p.?	Enchancer	[21]
P63	*PTF1A*		g.23508363A>G/g.23508363A>G	p.?/p.?	Enchancer	[22]
P64	*PTF1A*		g.23508363A>G/g.23508363A>G	p.?/p.?	Enchancer	
P65	*PTF1A*		g.23508363A>G/g.23508363A>G	p.?/p.?	Enchancer	
P66	*PTF1A*		g.23508363A>G/g.23508363A>G	p.?/p.?	Enchancer	
P67	*PTF1A*		g.23508363A>G/g.23508363A>G	p.?/p.?	Enchancer	
P68	*PTF1A*		g.23508363A>G/g.23508363A>G	p.?/p.?	Enchancer	
P69	*PTF1A*		g.23508363A>G/g.23508363A>G	p.?/p.?	Enchancer	
P70	*PTF1A*		g.23508363A>G/g.23508363A>G	p.?/p.?	Enchancer	
P71	*PTF1A*		g.23508365A>G/g.23508365A>G	p.?/p.?	Enchancer	
P72	*PTF1A*		g.23508365A>G/g.23508446A>C	p.?/p.?	Enchancer	[21]
P73	*PTF1A*		g.23508437A>G/g.23508437A>G	p.?/p.?	Enchancer	[21]
P74	*PTF1A*		g.23508437A>G/g.23508437A>G	p.?/p.?	Enchancer	[21]
P75	*PTF1A*		g.23508437A>G/g.23508437A>G	p.?/p.?	Enchancer	[21]
P76	*PTF1A*		g.23508437A>G/g.23508437A>G	p.?/p.?	Enchancer	[21]
P77	*PTF1A*		g.23508437A>G/g.23508437A>G	p.?/p.?	Enchancer	[21]
P78	*PTF1A*		g.23508437A>G/g.23508437A>G	p.?/p.?	Enchancer	[21]
P79	*PTF1A*		g.23508437A>G/g.23508437A>G	p.?/p.?	Enchancer	[22]
P80	*PTF1A*		g.23508437A>G/g.23508437A>G	p.?/p.?	Enchancer	
P81	*PTF1A*		g.23508437A>G/g.23508437A>G	p.?/p.?	Enchancer	
P82	*PTF1A*		g.23508437A>G/g.23508437A>G	p.?/p.?	Enchancer	
P83	*PTF1A*		g.23508437A>G/g.23508437A>G	p.?/p.?	Enchancer	
P84	*PTF1A*		g.23508437A>G/g.23508437A>G	p.?/p.?	Enchancer	
P85	*PTF1A*		g.23508437A>G/g.23508437A>G	p.?/p.?	Enchancer	
P86	*PTF1A*		g.23508437A>G/g.23508437A>G	p.?/p.?	Enchancer	
P87	*PTF1A*		g.23508437A>G/g.23508437A>G	p.?/p.?	Enchancer	
P88	*PTF1A*		g.23508437A>G/g.23508437A>G	p.?/p.?	Enchancer	
P89	*PTF1A*		g.23508437A>G/g.23508437A>G	p.?/p.?	Enchancer	
P90	*PTF1A*		g.23508437A>G/g.23508437A>G	p.?/p.?	Enchancer	
P91	*PTF1A*		g.23508437A>G/g.23508437A>G	p.?/p.?	Enchancer	
P92	*PTF1A*		g.23508437A>G/g.23508437A>G	p.?/p.?	Enchancer	
P93	*PTF1A*		g.23508437A>G/g.23508437A>G	p.?/p.?	Enchancer	
P94	*PTF1A*		g.23508437A>G/g.23508437A>G	p.?/p.?	Enchancer	
P95	*PTF1A*		g.23508437A>G/g.23508437A>G	p.?/p.?	Enchancer	
P96	*PTF1A*		g.23508437A>G/g.23508437A>G	p.?/p.?	Enchancer	
P97	*PTF1A*		g.23508437A>G/g.23508437A>G	p.?/p.?	Enchancer	
P98	*PTF1A*		g.23508437A>G/g.23508437A>G	p.?/p.?	Enchancer	
P99	*PTF1A*		g.23508437A>G/g.23508437A>G	p.?/p.?	Enchancer	
P100	*RFX6*	NM_173560.3	c.541C>T/c.541C>T	p.(Arg181Trp)/p.(Arg181Trp)	Exon 4	[23]
P101	*RFX6*	NM_173560.3	c.1573C>T/c.1573C>T	p.(Arg525*)/p.(Arg525*)	Exon 15	

Table S1. Pathogenic variants identified in the pancreatic agenesis cohort

Gene	gNomen	cNomen	pNomen	GnomAD Frequency	GnomAD pLI	GnomAD Z-sense (missense)
CNOT1	Chr16(GRCh37):g.58610468G>A	NM_016284.3:c.1603C>T	p.(Arg535Cys)	0%	1	7.62
SCAP	Chr3(GRCh37):g.47469072C>A	NM_012235.3:c.496G>T	p.(Glu166*)	0%	0	2.69
ZNF189	Chr9(GRCh37):g.104171066C>G	NM_003452.2:c.1016C>G	p.(Thr339Ser)	0%	0	0.51

Table S2. ***De novo*** **coding variants identified in P03**

ID	Genotype	Allele Depth	Read Depth
**P01**	0/1	28,38	63
**P02**	0/1	32,42	74
**P03**	0/1	30,15	45

Table S3. Exome sequencing allele and read depth of the NM_016284.4(*CNOT1*):c.1603C>T, p.(Arg535Cys) variant

ID	P01	P02[24]	P03
Birth weight (g)/ Gestation (weeks)	1340gr/39 wks (Z score=-3.5)	1100gr/38 wks (Z score=-3.8)	1900gr/39 wks (Z score=-2.4)
Gender	Female	Female	Male
CNOT1 Mutation	p.(Arg535Cys)	p.(Arg535Cys)	p.(Arg535Cys)
De novo	Maternal Sample N/A	Yes	Yes
Exome sequencing strategy	Singleton	Singleton	Trio
Age at last assessment (years)	11	Died at 12 weeks	18
Diabetes	Yes	Yes	Yes
Age at diabetes diagnosis	1 day	1 day	13 weeks
Insulin dose	On insulin pump (dose unknown)	N/A	0.9 u/kg/d
HbA1c	6.90%	N/A	8.30%
Pancreatic agenesis	Yes, (MRI)	Yes, (MRI and post mortem)	Abdominal CT
Exocrine pancreatic insufficiency	Yes, Creon treated	Yes, Creon treated	Yes, Creon treated
Gallbladder agenesis	Yes	Yes	
Neurological features	Lobular holoprosencephaly with dysplastic frontal horns of the lateral ventricles, missing septum pellucidum, broadly joined cella media of the lateral ventricles, malformation of the corpus callosum (only present in the rostral portion of the corpus and the splenium, the further frontal parts are not present). Complex focal seizures diagnosed aged 3 years.	Semi-lobar holoprosencephaly with polymicrogyria and fusion of the frontal lobes, absent corpus callosum	N/A
Developmental delay	Yes (mild), mainstream school at the age of 9 years with an accompanying person	N/A	No
Dysmorphic features	Low-set ears	Receding forehead, cylindrical nose, mild hypotelorism, dysplastic left ear, hypoplastic zygomatic bone	Craniofacial anomalies (prominent occiput, low-set ears, high arched palate, prominent central incisors)
Additional features	Transient elevation of LFTs, on growth hormone therapy	Thumbs in abduction at birth	Transient elevation of LFTs

Table S4. Clinical features of the patients with the *CNOT1* p.(Arg535Cys) mutation

Sex	Male		Female	
Genotype	Wildtype	Heterozygote	Wildtype	Heterozygote
Number	218	127	189	136
Percent	32%	19%	28%	20%

Table S5. Frequency of sex and genotype from wild type x heterozygote matings.

Genotype	Wildtypes		Heterozygotes		Homozygotes	
**Phenotype**	Number of embryos	**Percent**	**Number of embryos**	**Percent**	**Number of embryos**	**Percent**
**Exencephaly**					12	35.3
**Spina bifida**			1	1.4	3	8.8
**Eye defect**					3	8.8
**Coloboma**			1	1.4	10	29.4
**Eye missing**			2	2.7		
**Oedema**	2	4.4	11	14.9	19	55.9
**Bloody gut sac**	1	2.2	1	1.4		
**Midline defect**			3	4.1	1	2.9
**Smaller**	1	2.2	1	1.4	1	2.9
**Growth retarded**	3	6.7	2	2.7	2	5.9
**Webbed feet**	1	2.2			1	2.9
**Kinky tail**			1	1.4		
**Dark yolk sac**	1	2.2	1	1.4		
**Spine wrong shape**	1	2.2				
**Odd snout shape**					1	2.9
**NORMAL APPEARANCE**	35	77.8	57	77.0	8	23.5
**TOTALS**	45		74		34	

Table S6. Gross external phenotypes observed in E14.5 embryos

Supplemental Methods

Subjects. Individuals with pancreatic agenesis (defined as neonatal diabetes requiring insulin, and exocrine pancreatic insufficiency requiring oral enzyme replacement) were recruited by their clinicians for molecular genetic analysis in the Exeter Molecular Genetics Laboratory. The study was conducted in accordance with the Declaration of Helsinki, and all subjects or their parents gave informed consent for genetic testing. REC (Research Ethic Committee) reference: 17/WA/0327.

Exome sequencing. Exonic sequences were enriched from genomic DNA using Agilent's SureSelect Human All Exon kit (version 4) and then sequenced on an Illumina HiSeq 2000 sequencer using 100-bp paired-end reads. We used BWA (v0.6.2)5 to align sequence reads to the hg19 reference genome and GATK (v2.2-10) to call SNVs and indels.

Sanger sequencing confirmation. We amplified *CNOT1* exon 14 (NM_016284) using in-house designed primers (F – TGGCTTTCCCATAAAGAACG, R – TTCACCATGTTTTGGTCAGG). PCR products were sequenced on an ABI3730 capillary machine (Applied Biosystems) and analysed using Mutation Surveyor v3.98 (SoftGenetics). The bioinformatics tools SIFT, PolyPhen-2 and Align GVGD were accessed through the Alamut Visual software (Interactive Biosoftware) to predict the effect of the p.(Arg535Cys) variant *in silico*.

Mouse husbandry

All experiments were carried out in accordance with UK Home Office regulations, UK Animals (Scientific Procedures) Act 1986 and with approval from Wellcome Sanger Institute's Animal Welfare Committee. All mice were maintained in specific pathogen free facilities in individually ventilated cages at standard temperature (19-23°C) and humidity (55% ±10%), on a 12h dark, 12h light cycle. Mice were fed breeder's chow (LabDiets 5021, LabDiet, London, UK).

Cnot1$^{p.(Arg535Cys)}$ **mouse generation**

A one-step CRISPR/Cas9 homology-directed repair approach was used to generate *Cnot1*$^{p.(Arg535Cys)}$-mutant mice. C57BL/6N mouse zygotes were injected with *in vitro* transcribed Cas9 mRNA, single guide RNA targeting exon 14 of the *Cnot1* gene and a 126 bp single-stranded oligonucleotide (Ultramer DNA oligos, Integrated DNA Technologies) complementary to the Cas9 cleavage site. The oligonucleotide was designed to encode for the p.(ARG535CYS) substitution in *Cnot1* and harboured two additional silent mutations to limit DNA target recleavage by Cas9 after homology-directed repair. The embryos were transferred into the oviduct of pseudopregnant females the same day of microinjection. Correctly targeted, G0 mosaic offspring were identified by DNA sequencing of PCR products generated using primers CTGTAATTAATTGGTGCTTGAGCCTGACTG and CCATGTCTAAGTGCCTGATTCTGAATGCC which amplify *Cnot1* exon 14. G0 founder mice were mated to C57BL/6N mice to establish G1s for further breeding to C57BL/6N mice.

Cnot1 sgRNA ATTATAAGTTGGCGAATTGA

Cnot1 $^{p.(Arg535Cys)}$-repair oligonucleotide (nucleotide substitutions in lower case)
TGGCTAACATGGTTCCTGTTTAATGTTCCCTCCTCTTTTCTTAGGGGCAGTCTCCATCgATctGCCAACTTATAAT
GCATGCAATGGCAGAATGGTACATGAGAGGGGAGCAGTATGATCAGGCCA

Genotyping

Genotyping was performed on DNA extracted from ear biopsies (adults) or yolk sac (embryos). DNA was extracted by HotSHOT[1] and genotyping performed using the LGC KASP™ system (LGC,

Teddington, UK)[2]. Plates were read using a PHERAstar plus plate reader (BMG LABTECH, Offenburg, Germany) and genotypes determined using the software KlusterCaller (LGC).

Embryo collection

For gross morphology imaging, embryos were harvested at E14.5 and dissected in cold pH7.4 PBS. They were scored for gross abnormalities and imaged using a Leica M205C microscope, DFC495 camera and LAS v4 software.

HREM

For HREM, embryos were harvested at E14.5 and dissected in 37°C HBSS (no calcium, magnesium or phenol red, LifeTech) supplemented with EDTA (0.01M LifeTech). Embryos were exsanguinated by severing of the umbilical cord and rocking in warm buffer for approximately 20 minutes, before fixation in Bouin's solution for 24 h. Embryos were stored in PBS supplemented with 0.01% sodium azide between fixation and HREM processing.

Embryos were dehydrated in methanol and embedded in methacrylate resin (JB-4, PolySciences) containing eosin B and acridine orange. Digital volume data generation was performed using high-resolution episcopic microscopy (HREM), as previously described[3-6]. Phenotype scoring was performed using virtual 2D sections, with abnormalities classified using the Mammalian Phenotype (MP) ontology[7]. Developmental staging was determined from external morphology using SD volume rendered models as described by Greyer *et al*[8].

In the original axial HREM sections of 5 *Cnot1*$^{p.(Arg535Cys)}$ homozygous mutants (two of Geyer stage (GS) 22, two of GS22+, one of GS23-), 5 CNOT *Cnot1*$^{p.(Arg535Cys)}$ heterozygous mutants (one of GS22, four of GS22+) and 18 controls (six of each, GS22, GS22+, GS23-) the ductal tissues of the pancreas were segmented employing the software package Amira 5.4.5 (Thermo Fisher Sientific) and its interactive thresholding and manual tracing tools. Separate 3D surface models of the ventral and dorsal pancreas were created and the volumes of the 3D models were determined using the "MaterialStatistics" tool of Amira. The results were statistically analysed using Excel for Mac 2011.

RNA extraction & qPCR

For pancreas dissection, embryos were harvested at E14.5 and dissected in cold pH7.4 PBS. The pancreas was removed and placed in RNAlater at 4°C for 24 h, before storage at -20°C. Tissue was homogenised in Trizol using a Qiagen TissueLyser, and RNA extracted using chloroform followed by the Qiagen MinElute kit (Qiagen, Hilden, Germany). RNA concentration and purity was evaluated by NanoDrop (Thermo Scientific, Wilmington, DE, USA). RNA integrity was further assessed using an Agilent Bioanalyzer (Agilent Technologies, Santa Clara, CA, USA). cDNA synthesis was performed using 200 ng RNA, random primers and Superscript II reverse transcriptase (Life Technologies, Carlsbad, CA, USA). qPCR was performed using Sybr Green (Applied Biosystems, Foster City, CA, USA) and run on an AB7500 qPCR machine (Applied Biosystems). The primers used were as follows:

**Gene**	**Forward Primer**	**Reverse Primer**
18S	GTAACCCGTTGAACCCCATT	CCATCCAATCGGTAGTAGCG
Gapdh	TGGTTCACACCCATCACAAACA	GGTGAAGGTCGGTGTGAACGG
Hnf1b	CAAGATGTCAGGAGTGCGCTA	CTCCCGACACTGTGATCTGC
Insulin	TGGCTTCTTCTACACACCCAAG	ACAATGCCACGCTTCTGCC
Pdx1	CAGTGGGCAGGAGGTGCTTA	CCAGATTTTGATGTGTCTCTCGG
Prss1 (Typsin-1)	GCTGACTGTGAGGCTTCCTA	AGAGTCACCCTGGCAGGAAT

Ptf1a	CATCGAGGCACCCGTTCA	GTCCAGGAAAGAGAGTGCCC
Rpl32	GGCCAGATCCTGATGCCCAAC	CAGCTGTGCTGCTCTTTCTAC
Shh	TTCCCAACGTAGCCGAGAAG	TTCCCAACGTAGCCGAGAAG

Relative expression was calculated using the ΔΔCt method, relative to the cubic mean of three reference genes (*18S*, *Gapdh* and *Rpl32*)[9].

Statistics

Genotype frequency data were analysed using Chi-Squared. All other data analysis was performed in R.

Embryo phenotype data were analysed using the Fisher's exact test assuming an additive model. Pancreas size and qPCR data were analysed using ANOVA with TukeyHSD post hoc test, including stage number as a co-variate when analysing pancreas size.

Data availability

All HREM data from this study will be made available on the DMDD website (https://dmdd.org.uk).

Supplemental References

1. Truett, G.E. *et al.* Preparation of PCR-quality mouse genomic DNA with hot sodium hydroxide and tris (HotSHOT). *Biotechniques* **29**, 52, 54 (2000).
2. Cuppen, E. Genotyping by Allele-Specific Amplification (KASPar). *CSH Protoc* **2007**, pdb.prot4841 (2007).
3. Mohun, T.J. & Weninger, W.J. Imaging heart development using high-resolution episcopic microscopy. *Curr Opin Genet Dev* **21**, 573-8 (2011).
4. Mohun, T.J. & Weninger, W.J. Embedding embryos for high-resolution episcopic microscopy (HREM). *Cold Spring Harb Protoc* **2012**, 678-80 (2012).
5. Weninger, W.J. *et al.* High-resolution episcopic microscopy: a rapid technique for high detailed 3D analysis of gene activity in the context of tissue architecture and morphology. *Anat Embryol (Berl)* **211**, 213-21 (2006).
6. Mohun, T.J. & Weninger, W.J. Generation of volume data by episcopic three-dimensional imaging of embryos. *Cold Spring Harb Protoc* **2012**, 681-2 (2012).
7. Weninger, W.J. *et al.* Phenotyping structural abnormalities in mouse embryos using high-resolution episcopic microscopy. *Dis Model Mech* **7**, 1143-52 (2014).
8. Geyer, S.H. *et al.* A staging system for correct phenotype interpretation of mouse embryos harvested on embryonic day 14 (E14.5). *J Anat* **230**, 710-719 (2017).
9. Livak, K.J. & Schmittgen, T.D. Analysis of relative gene expression data using real-time quantitative PCR and the 2(-Delta Delta C(T)) Method. *Methods* **25**, 402-8 (2001).
10. Shaw-Smith, C. *et al.* GATA4 mutations are a cause of neonatal and childhood-onset diabetes. *Diabetes* **63**, 2888-94 (2014).
11. Allen, H.L. *et al.* GATA6 haploinsufficiency causes pancreatic agenesis in humans. *Nat Genet* **44**, 20-22 (2011).
12. McMillan, T., Girgis, R. & Sellers, E.A. Neonatal diabetes and protein losing enteropathy: a case report. *BMC Med Genet* **17**, 32 (2016).
13. De Franco, E. *et al.* GATA6 mutations cause a broad phenotypic spectrum of diabetes from pancreatic agenesis to adult-onset diabetes without exocrine insufficiency. *Diabetes* **62**, 993-7 (2013).
14. Savova, R. *et al.* Marked intrafamilial variability of exocrine and endocrine pancreatic phenotypes due to a splice site mutation in GATA6. *Biotechnology & Biotechnological Equipment* **32**, 124-129 (2018).
15. Ellard, S. *et al.* Improved genetic testing for monogenic diabetes using targeted next-generation sequencing. *Diabetologia* **56**, 1958-63 (2013).
16. Catli, G. *et al.* A novel GATA6 mutation leading to congenital heart defects and permanent neonatal diabetes: a case report. *Diabetes Metab* **39**, 370-4 (2013).
17. De Franco, E. *et al.* Biallelic PDX1 (insulin promoter factor 1) mutations causing neonatal diabetes without exocrine pancreatic insufficiency. *Diabet Med* **30**, e197-200 (2013).
18. Flanagan, S.E. *et al.* Analysis of transcription factors key for mouse pancreatic development establishes NKX2-2 and MNX1 mutations as causes of neonatal diabetes in man. *Cell Metab* **19**, 146-54 (2014).
19. Gabbay, M., Ellard, S., De Franco, E. & Moises, R.S. Pancreatic Agenesis due to Compound Heterozygosity for a Novel Enhancer and Truncating Mutation in the PTF1A Gene. *J Clin Res Pediatr Endocrinol* **9**, 274-277 (2017).
20. Houghton, J.A. *et al.* Isolated Pancreatic Aplasia Due to a Hypomorphic PTF1A Mutation. *Diabetes* **65**, 2810-5 (2016).
21. Weedon, M.N. *et al.* Recessive mutations in a distal PTF1A enhancer cause isolated pancreatic agenesis. *Nat Genet* **46**, 61-64 (2014).
22. Evliyaoglu, O. *et al.* Neonatal Diabetes: Two Cases with Isolated Pancreas Agenesis due to Homozygous PTF1A Enhancer Mutations and One with Developmental Delay, Epilepsy, and

Neonatal Diabetes Syndrome due to KCNJ11 Mutation. *J Clin Res Pediatr Endocrinol* **10**, 168-174 (2018).

23. Zegre Amorim, M. *et al.* Mitchell-Riley Syndrome: A Novel Mutation in RFX6 Gene. *Case Rep Genet* **2015**, 937201 (2015).
24. Hilbrands, R. *et al.* Pancreas and gallbladder agenesis in a newborn with semilobar holoprosencephaly, a case report. *BMC Med Genet* **18**, 57 (2017).
